# The TGFα-EGFR-Akt signaling axis plays a role in enhancing proinflammatory chemokines in triple-negative breast cancer cells

**DOI:** 10.18632/oncotarget.25389

**Published:** 2018-06-29

**Authors:** Rosa Mistica C. Ignacio, Carla R. Gibbs, Eun-Sook Lee, Deok-Soo Son

**Affiliations:** ^1^ Department of Biochemistry and Cancer Biology, Meharry Medical College, Nashville, TN 37208 USA; ^2^ Department of Pharmaceutical Sciences, College of Pharmacy, Florida A&M University, Tallahassee, FL 32301 USA

**Keywords:** proinflammatory chemokine, Akt, EGFR, TGFα, triple-negative breast cancer

## Abstract

Triple-negative breast cancer (TNBC) is aggressive and typically has a poor prognosis. Chemokines have chemoattractant potential for cancer metastasis. Here, we investigated the chemokine signatures in BC subtypes and the underlying mechanisms that enhance proinflammatory chemokines in TNBC. Analysis from microarray dataset revealed that basal-like BC subtype including TNBC expressed dominantly proinflammatory chemokines, such as CXCL1 and 8, compared to non-TNBC. Chemokine PCR array confirmed the dominant chemokines in TNBC cells. To identify a driving factor for proinflammatory chemokines in TNBC cells, we determined the expression and signaling profiles of epidermal growth factor receptor (EGFR) family members. TNBC cells expressed higher levels of EGFR and phosphorylated Akt/Erk than non-TNBC cells. In addition, EGF further enhanced the proinflammatory chemokines in TNBC cells, including CXCL2. Knockdown of Akt reduced the CXCL2 promoter activity, while overexpression of Akt enhanced it. MK2206, an Akt inhibitor, reduced the CXCL2 promoter activity, while inhibition and knockdown of Erk did not reduce its activity. We found that transforming growth factor alpha (TGFα) could serve as a main ligand for EGFR to drive EGFR-mediated Akt activation in TNBC cells. MK2206 decreased TGFα promoter activity, while overexpression of Akt increased it. MK2206 also reduced TGFα release from TNBC cells. Moreover, MK2206 downregulated CXCL2 mRNA expression, while TGFα upregulated it. Taken together, the TGFα-EGFR-Akt signaling axis can play a role in enhancing proinflammatory chemokine expression in TNBC, subsequently contributing to the inflammatory burden that ultimately lead to cancer progression and a higher mortality rate among TNBC patients.

## INTRODUCTION

Breast cancer (BC) is the most frequently diagnosed cancer and the second most common cause of cancer deaths in women in the US [1]. Gene expression profiling has classified BC into four distinct intrinsic molecular subtypes as follows: luminal A (LA), luminal B (LB), epidermal growth factor receptor 2 (HER2)-enriched, and basal-like (BL) BC including triple-negative breast cancer (TNBC) [2–5]. The LA subtype is estrogen receptor (ER)- and progesterone receptor (PR)-positive, and typically has a good prognosis [5]. On the other hand, accounting for 15–20% of total BC, TNBC is more aggressive and typically has a poor prognosis [6]. TNBC cells have little or no response to many current treatments for BC due to the cancer heterogeneity and the absence of a well-defined therapeutic target, such as ER, PR, and HER2. Apart from being aggressive, TNBC tumors grow bigger, are of higher grade, and often present lymph node involvement [7–9]. While TNBC patients generally respond well to neoadjuvant chemotherapy, they still face a higher rate of metastasis compared to patients with other subtypes of BC; a five-year survival for women with metastatic TNBC is less than 30% [7, 10]. Therefore, treating TNBCs remains a major challenge.

TNBCs are heterogenous because these tumors are comprised of several molecular subtypes with discrete histological features and oncogenic signaling pathways [11–13]. Recent molecular analysis defined TNBC into four subtypes, namely basal-like 1 and 2 (BL1, BL2), mesenchymal-like (ML) and luminal androgen (LAR) [14]. ML-TNBC accounts for 30% of TNBCs and has worse prognosis due to enhanced epithelial-to-mesenchymal transition (EMT) [11, 15] and elevated expression of genes involved in growth factor pathways [14]. BL1-TNBC presents with high levels of cell cycle aberrations and DNA damage, while BL2-TNBC shows enhanced activation of growth factor pathways [11, 16]. LAR-TNBC is driven by androgen signaling [11].

To better understand the molecular basis of TNBC, we need to identify its molecular drivers for the aggressiveness. Considerable attention has recently been paid to the possible involvement of chemokines and chemokine receptors in BC progression and metastasis [17]. Chemokines, small cytokine-like proteins that interact with G-protein-coupled receptors, play a role in cell proliferation, inflammation, invasion, metastasis, angiogenesis, and tumorigenesis, in addition to regulating immune cell migration during immune responses [18–23]. The CXC chemokine family are implicated in both angiogenesis and anti-angiogenesis [23]. Proinflammatory chemokines such as CXCL1, 2, 3, 5, 6 and 8 interact with the CXCR2 receptor and can mediate angiogenesis during cancer development [23, 24]. Normal breast tissues express these chemokines at relatively low levels [17]. On the other hand, TNBC tissues have a higher proinflammatory index than non-TNBC tissues. Altered expression of transcription factors such as NF-κB affects chemokine levels and may promote tumorigenesis [25, 26]. In this study, we investigated the chemokine signatures in TNBC and non-TNBC cells and elucidated the underlying mechanisms that enhance proinflammatory chemokine expression in TNBC.

## RESULTS

### BL- and ML-TNBC cells express high levels of proinflammatory chemokines

**Figure 1:**
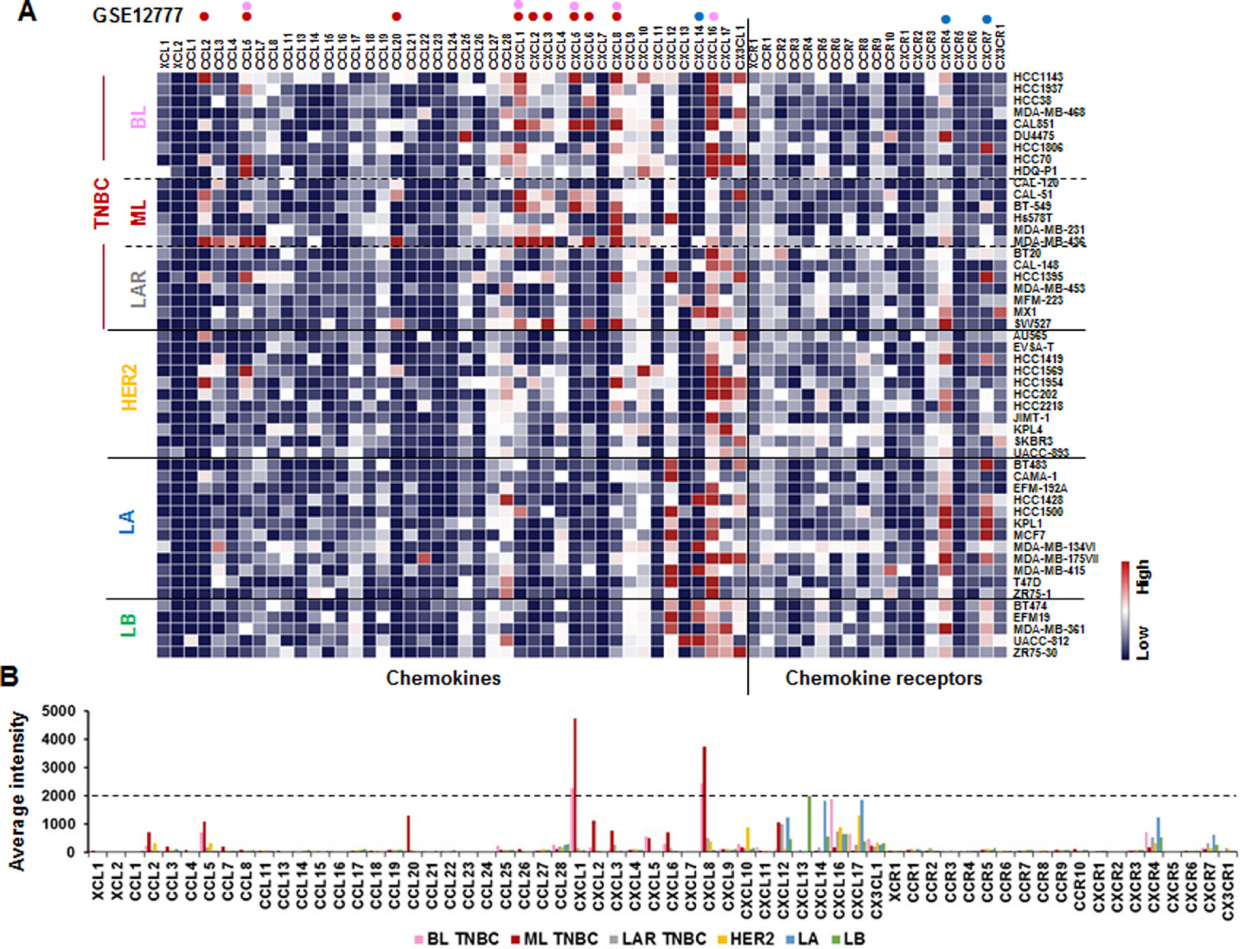
Chemokine signatures in BC cell lines (**A**) Heatmap for RNA expression levels of chemokines and chemokine receptors based on analysis of GEO dataset (Accession: GSE12777) with 51 human BC cell lines using Gitools 2.3.1. Pink and red dots indicate high expression levels in basal-like (BL)- and mesenchymal-like (ML)-TNBC cells, respectively. Blue dots indicate high expression levels in luminal A (LA) subtype. LAR: luminal androgen receptor, LB: luminal B. (**B**) Average intensity for the expression levels of dominant chemokines and chemokine receptors in different BC subtypes. Pink, red, grey, yellow, blue, and green bars indicate BL-TNBC, ML-TNBC, LAR-TNBC, HER2, LA and LB subtypes, respectively.

**Figure 2:**
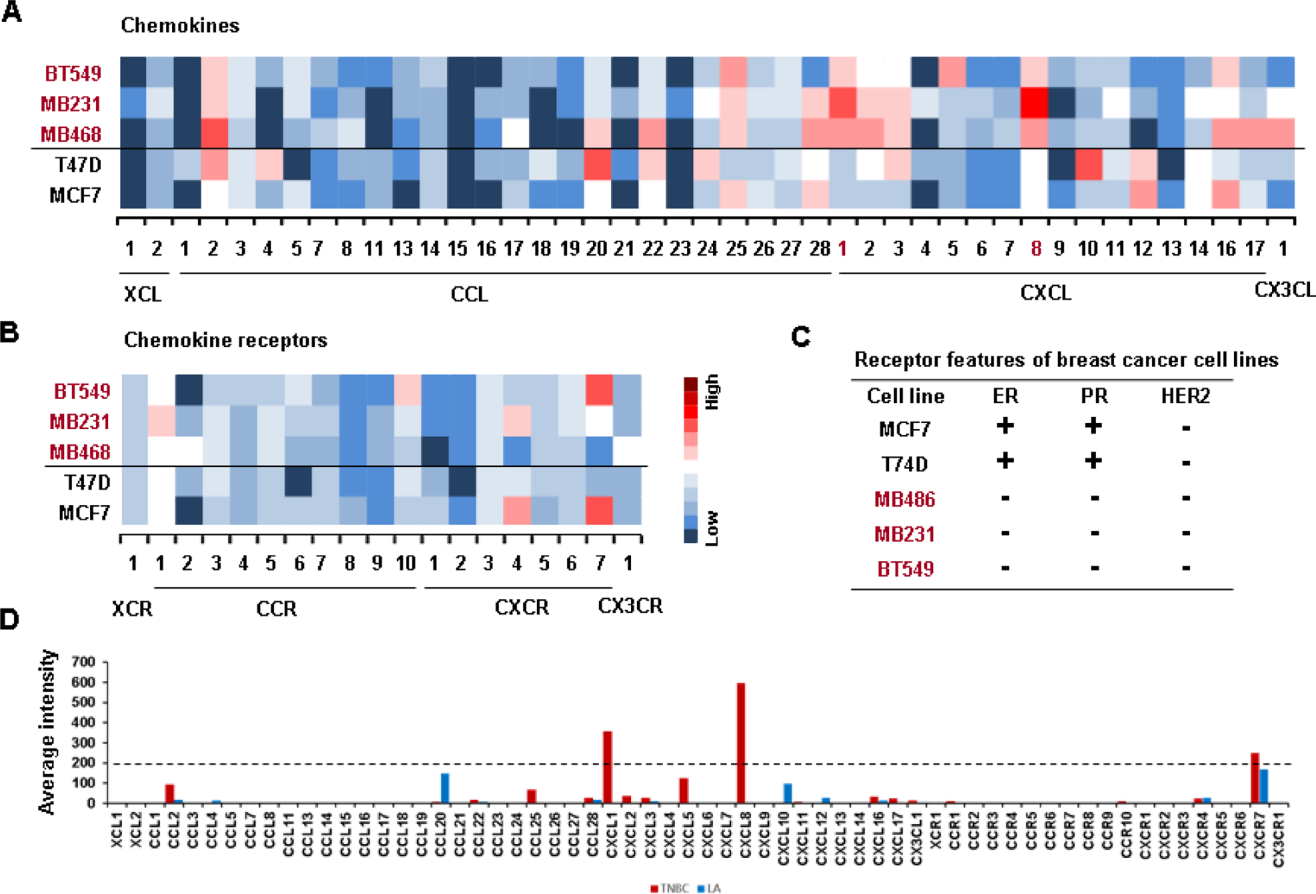
Confirmation of PCR array on elevated proinflammatory chemokines in TNBC cells (**A**) Heatmap for RNA expression levels of chemokines in representative human TNBC (BT549, MB231 and MB468) and non-TNBC (T47D, MCF7) cell lines. (**B**) Heatmap for RNA expression levels of chemokine receptors in TNBC and non-TNBC cell lines. After isolating total RNA and choosing the qualified RNAs, a human chemokine PCR array was performed. Red trend indicates high expression levels of chemokines. (**C**) The status of estrogen receptor (ER), progesterone receptor (PR) and HER2 expression in TNBC and LA-BC cells. (**D**) Average intensity for the expression levels of dominant chemokines and chemokine receptors in TNBC and LA-BC cells. Red and blue bars indicate TNBC and LA-BC cells, respectively.

We investigated the chemokine signatures in human BC cancer lines to exclude the tumor heterogeneity observed in tumor tissues. Analysis of the National Center for Biotechnology Information (NCBI) Gene Expression Omnibus (GEO) dataset on 51 human BC cell lines revealed the following chemokine signatures: CXCL1, 5, 8 and 16 in BL-TNBC cells; CCL2, 5, 20, CXCL1, 2, 3, 5, 6 and 8 in ML-TNBC cells; and CXCL14, CXCR4 and 7 in LA cells (Figure 1A). Particularly, BL- and ML-TNBC cells dominantly expressed proinflammatory chemokines CXCL1 and 8, as the main CXCR2 ligands, compared to non-TNBC cells (Figure 1B). On the other hand, LAR-TNBC cells expressed lower levels of proinflammatory chemokines than BL- and ML-TNBC cells, being similar to non-TNBC cells (Figure 1A and 1B). We selected representative human TNBC (MB468, MB231 and BT549) and LA-BC (MCF7, T47D) cell lines to perform PCR array for 43 chemokines and 19 chemokine receptors. A web-based PCR array data analysis protocol provided by SABiosciences (Qiagen) defined >35, 30–35 and <30 average threshold cycles as absent, low expression level, and high expression level, respectively. BT549 cells expressed high levels of chemokines CCL2 and 25, CXCL1, 5, 8 and 16 (Figure 2A), and chemokine receptors CCR10 and CXCR7 (Figure 2B). MB231 cells expressed high levels of chemokines CCL2, 25 and 28, CXCL1, 2, 3 and 8 (Figure 2A), and chemokine receptors CCR1 and CXCR4 (Figure 2B). MB468 cells expressed high levels of chemokines CCL2, 20, 22, 25 and 28, CXCL1, 2, 3, 8, 16 and17, and CX3CL1 (Figure 2A). On the other hand, T47D cells expressed high levels of chemokines CCL2, 4, 20, 22 and 24, CXCL3, 10 and 12 (Figure 2A). MCF7 expressed high levels of chemokines CCL25 and 28, CXCL12 and 16 (Figure 2A), and chemokine receptors CXCR4 and 7 (Figure 2B). The comparison between the chemokine signatures in TNBC and LA-BC cells (Figure 2C) confirmed that TNBC cells dominantly expressed proinflammatory chemokines CXCL1 and 8 compared to LA-BC cells (Figure 2D).

### Basal-like BC subtype including TNBC has higher levels of proinflammatory chemokines than other BC subtypes in human BC tissues

**Figure 3:**
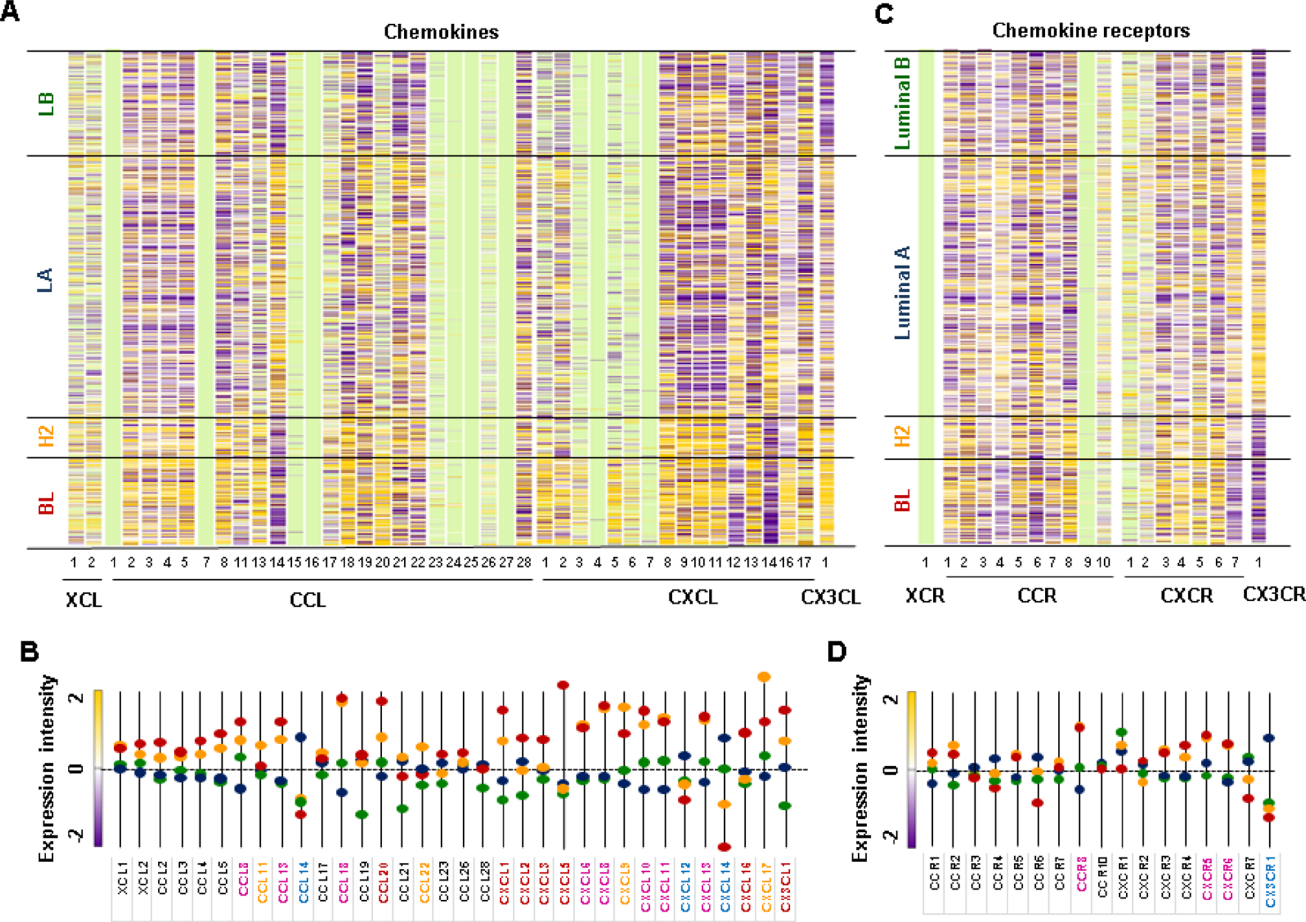
Chemokine signatures in human BC tissues (**A**) Heatmap for chemokine expression profiles in human BC tissues from TCGA-based dataset using Gitools 2.3.1. (**B**) Statistical analysis of chemokine expression profiles in human BC tissues. (**C**) Heatmap for chemokine receptor expression profiles in human BC tissues from TCGA-based dataset using Gitools 2.3.1 (**D**) Statistical analysis of chemokine receptor expression profiles in human BC tissues. BC tissues used include 140 basal-like (BL), 67 HER2 (H2), 419 Luminal A (LA), and 192 Luminal B (LB) samples. The bright green colors in (A) and (C) indicate no determination. The pink letters in (B) and (D) indicate dominant chemokines and chemokine receptors in both BL and HER2 samples. The red, yellow, and blue letters in (B) and (D) indicate dominant chemokines and chemokine receptors in BL, HER2, and LA samples, respectively. Significant increase (*p* ≤ 0.05) was determined using ANOVA and Tukey's pairwise comparisons.

We used The Cancer Genome Atlas (TCGA)-based dataset for human BC tissues to define the chemokine signatures in heterogenous breast tumors. Analysis of TCGA-based dataset using Gitools 2.3.1 revealed the following chemokine signatures: BL subtype representing TNBC expressed high levels of chemokines CCL20, CXCL1, 2, 3, 5 and 16, and CX3CL1 (Figure 3A and 3C); HER2 subtype expressed high levels of chemokines CCL11 and 22, CXCL9 and 17 (Figure 3A and 3C); LA subtype expressed high levels of chemokines CCL14, CXCL12 and 14 (Figure 3A and 3C) and chemokine receptor CX3CR1 (Figure 3B and 3D). Both BL and HER2 subtypes expressed high levels of chemokines CCL8, 13 and 18, CXCL6, 8, 10, 11 and 13 (Figure 3A and 3C), and chemokine receptors CCR8, CXCR5 and 6 (Figure 3B and 3D). The intersection between chemokine signatures in human BC cell lines and tissues (Figures 1 and 3) revealed that TNBC cells expressed high levels of proinflammatory chemokines CCL20, CXCL1, 2, 3, 5, 6, 8 and 16, while LA-BC cells expressed high levels of CXCL14 (Supplementary Figure 1).

### The BL-BC subtype has higher levels of EGFR compared to other BC subtypes in human BC tissues

**Figure 4:**
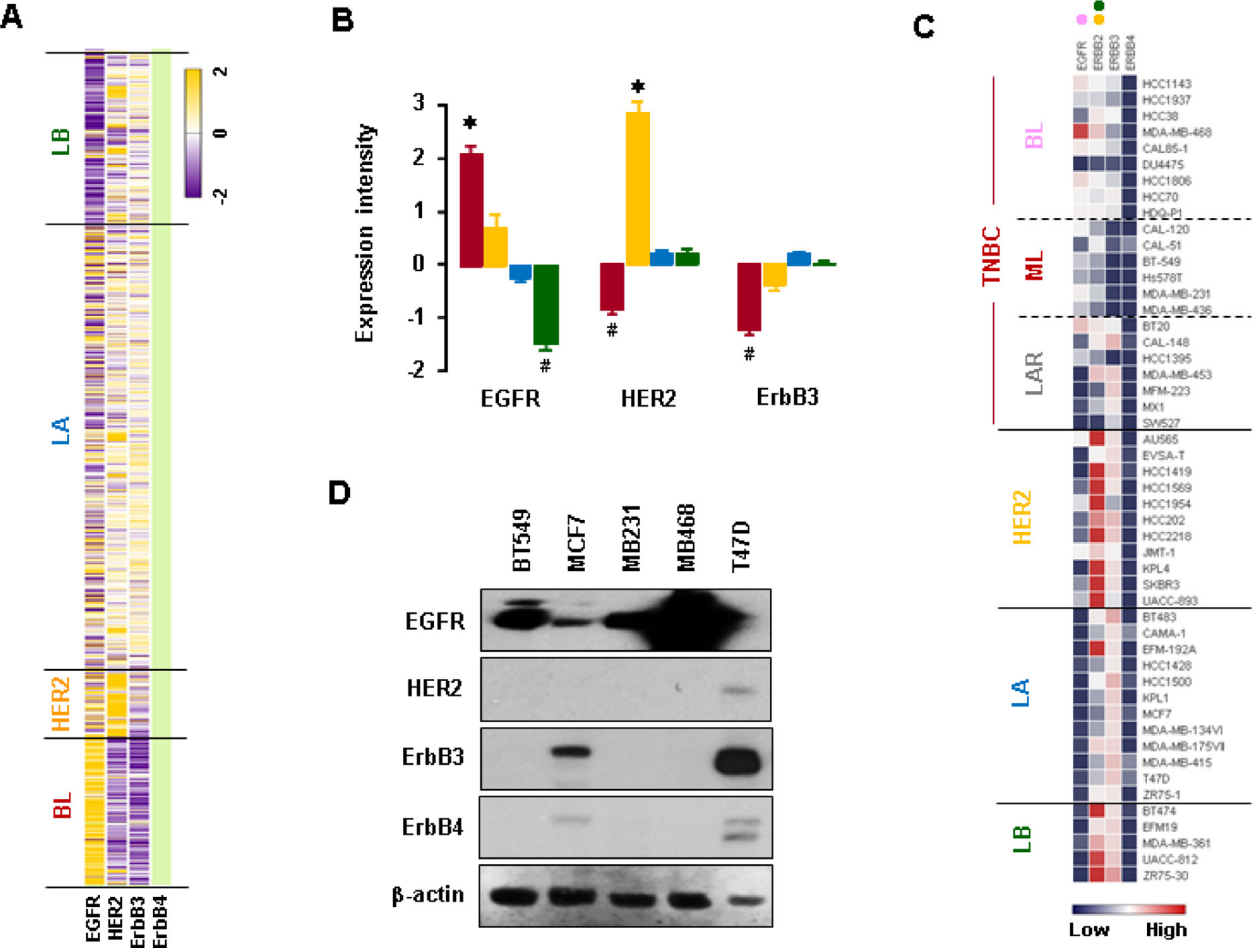
Expression profiles of EGFR family members in BC cells (**A**) Heatmap for RNA expression levels of EGFR family members in human BC tissues from TCGA-based dataset using Gitools 2.3.1. (**B**) Statistical analysis for RNA expression levels of EGFR family members in human BC tissues. The red, yellow, blue and green colors indicate BL, HER2, LA and LB samples, respectively. The asterisk (^*^) and hash (#) indicate a statistically significant increase and decrease (*p* ≤ 0.05) as calculated by ANOVA and Tukey's pairwise comparisons, respectively. (**C**) Heatmap for RNA expression levels of EGFR family members based on analysis of the GEO dataset (Accession: GSE12777) for 51 human BC cell lines using Gitools 2.3.1. Pink, yellow and green dots indicate high expression levels in BL-TNBC, HER2-BC and LB-BC cells, respectively. (**D**) Protein levels of EGFR family members in representative TNBC (MB468, MB231, and BT549) and non-TNBC (MCF7 and T47D) cells. β-actin was used as the loading control.

We analyzed expression profiles for EGFR family members using TCGA-based dataset. The BL-BC subtype representing TNBC expressed higher levels of EGFR mRNA than other subtypes such as LA-, LB- and HER2-BC (Figure 4A and 4B). On the other hand, HER2-BC subtype dominantly expressed HER2 mRNA, while BL-BC subtype expressed lower levels of ErbB3 mRNA (Figure 4A and 4B). Analysis of the NCBI GEO dataset on 51 human BC cell lines revealed that BL-TNBC cells had the highest expression levels of EGFR, while LA- and LB-BC subtypes had the lowest levels (Figure 4C and Supplementary Figure 2). HER2-BC and LB-BC cells expressed higher levels of HER2 than other types of BC cells, while BL- and ML-TNBC cells expressed lower levels of ErbB3 (Figure 4C and Supplementary Figure 2). LA-BC subtype cells expressed higher levels of ErbB4 than BL-TNBC cells (Figures 4C and Supplementary Figure 2). Moreover, western blot data also revealed that TNBC cells (MB468, MB231 and BT549) expressed higher levels of EGFR than non-TNBC cells (MCF7 and T47D), while non-TNBC cells expressed higher levels of ErbB3 and ErbB4 than TNBC cells (Figure 4D). In addition, we found that the elevation of CXCL1, 2, 5 and 8 has a positive correlation with EGFR (Supplementary Figure 3A), but not with HER2 and ErbB3 levels (Supplementary Figure 3B).

### EGF enhances proinflammatory chemokine expression in TNBC BT549 cells

**Figure 5:**
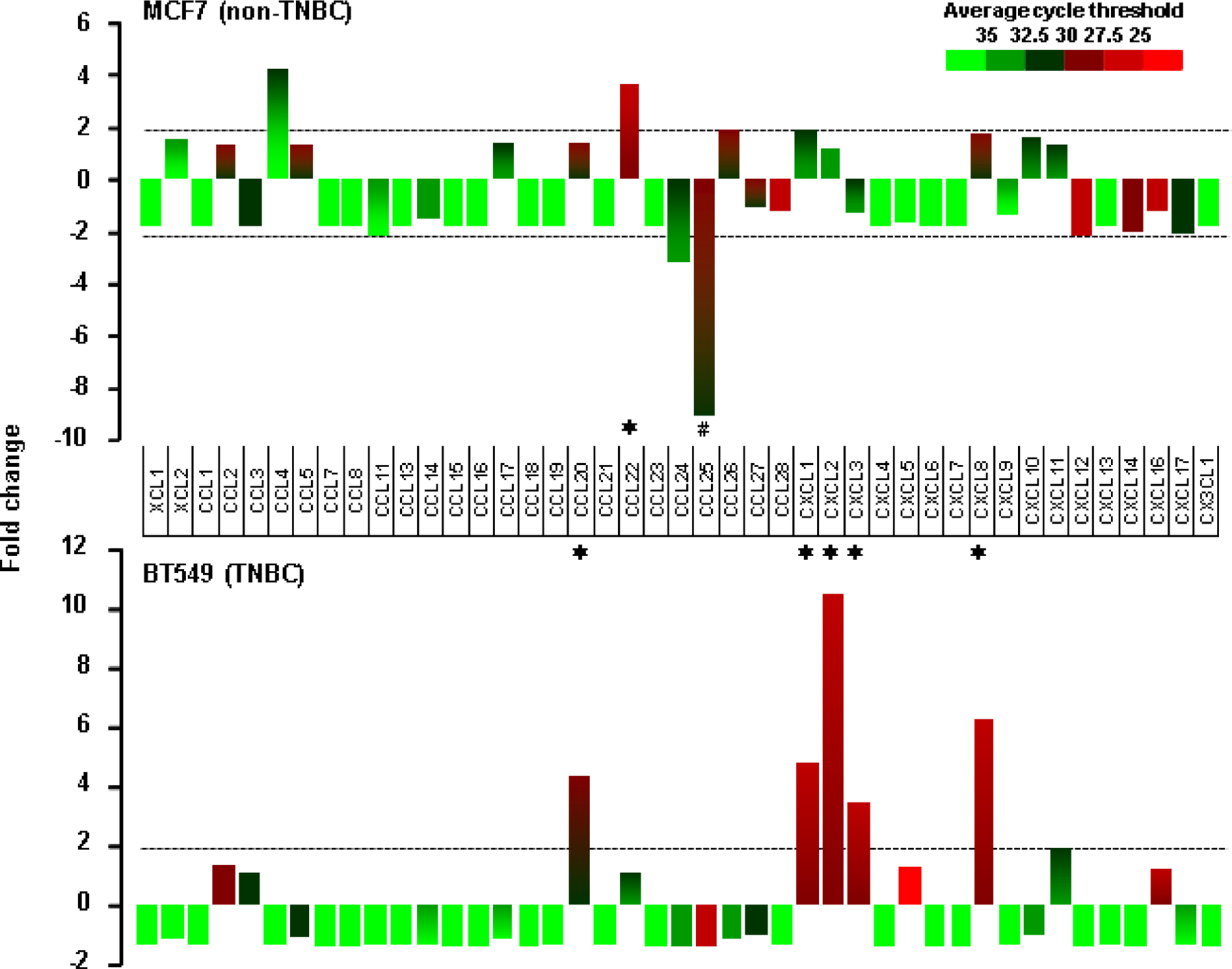
EGF-induced chemokines in non-TNBC and TNBC cells Chemokine signatures in non-TNBC (MCF7) and TNBC (BT549) cells after a 1-h stimulation with recombinant human EGF (10 ng/ml) as revealed by a human chemokine PCR array. Chemokines with duplicate average cycle threshold of <30 are considered dominant. The asterisk (^*^) and hash (^#^) indicate increases and decreases that are larger than two-fold, respectively.

**Figure 6:**
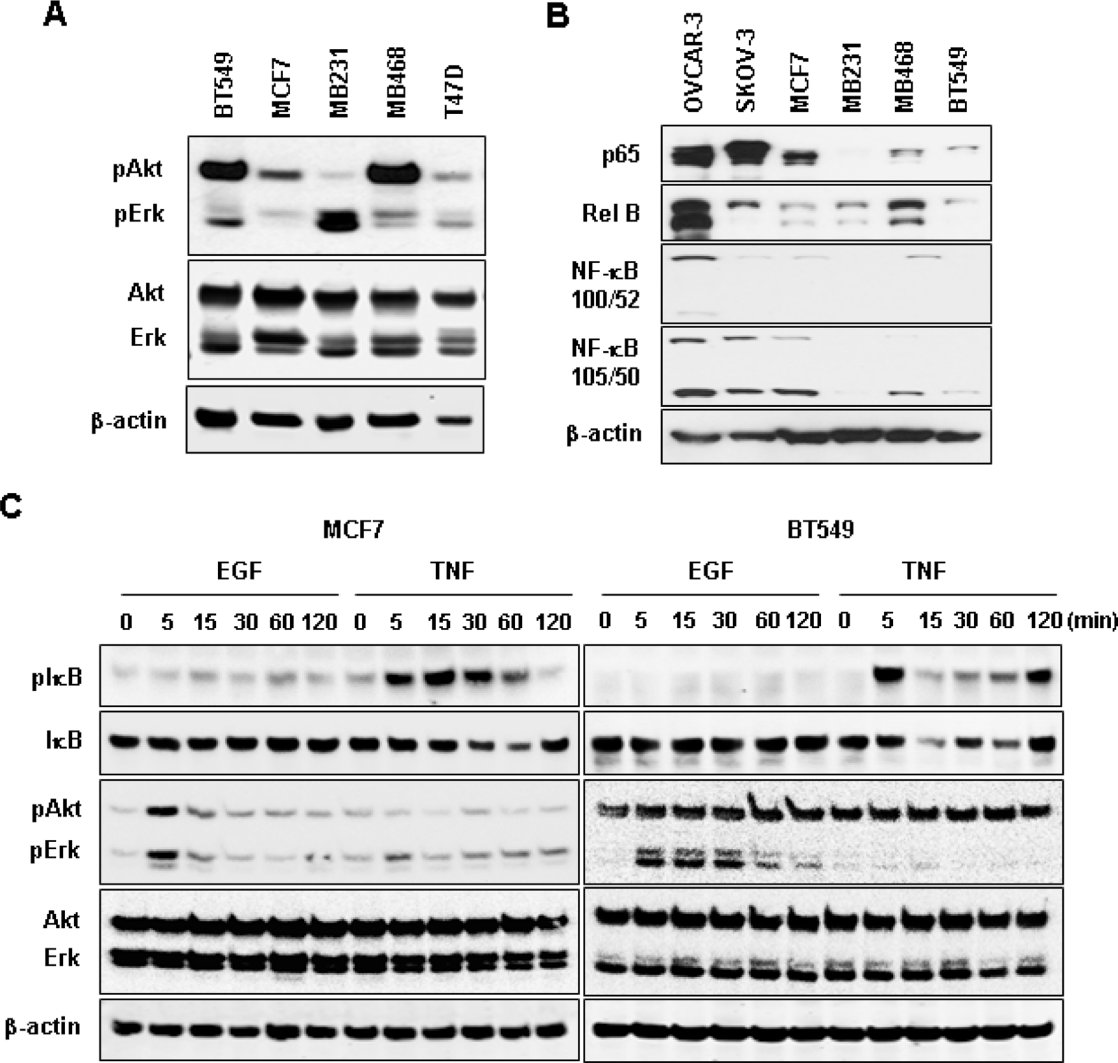
Protein expression profiles of Akt, Erk and NF-κB signaling in non-TNBC and TNBC cells (**A**) Basal protein levels of Akt and Erk in TNBC (BT549, MB231, MB468) and non-TNBC (MCF, T47D) cell lines. (**B**) Basal protein levels for NF-κB family members p65 (Rel A), Rel B, NF-κB (100/52), and NF-κB (105/50) in TNBC and non-TNBC cells. Human SKOV-3 and OVCAR-3 ovarian cancer cells were used as positive controls for the expression of NF-κB signaling components. (**C**) Protein expression profiles of IκB, Akt, Erk and their phosphorylated forms in BT549 and MCF7 cells in response to EGF (10 ng/ml) and TNF (10 ng/ml) treatments. β-actin was used as the loading control.

We selected MCF7 and BT549 as models for non-TNBC and TNBC cells, respectively, to identify EGF-responsive chemokines. After a 1-h stimulation with recombinant human EGF, BT549 cells showed more than two-fold induction in levels of CCL20, CXCL1, 2, 3 and 8, while MCF7 cells showed as increase in CCL22 levels and a decrease in CCL25 levels (Figure 5). The EGF exposure for 1 h had no effect on chemokine receptor expression in both cell types (data not shown). Based from the chemokine profiling of our representative cell lines under basal or unstimulated condition (Figure 2A), MB468 cells highly express CCL2 and CXCL2 compared to BT549 cells. However, when stimulated with EGF, MB468 cells showed only 3-fold induction of CXCL2 (Supplementary Figure 4) compared to above 10-fold induction in BT549 cells (Figure 5). Thus, for the subsequent mechanistic experiments, we selected BT549 cells as a model for TNBC. We also checked the status of downstream EGFR signaling components such as Akt, Erk and NF-κB in TNBC and non-TNBC cells at their basal levels. TNBC BT549 and MB468 cells expressed higher levels of phosphorylated Akt compared to non-TNBC MCF7 and T47D cells (Figure 6A). On the other hand, MB231 expressed higher levels of phosphorylated Erk (Figure 6A). We used human SKOV-3 and OVCAR-3 ovarian cancer cells as positive controls for NF-κB signaling components (Figure 6B) since those cells potently activated NF-κB [27]. TNBC and non-TNBC cells did not exhibit any difference in the basal levels of NF-κB signaling components (Figure 6B). Since EGF treatment produced different chemokine signatures in MCF7 and BT549 cells (Figure 5), we next compared the levels of EGF- and tumor necrosis factor α (TNF)-activated signaling pathways such as Akt, Erk and IκB in these cells. BT549 cells showed more activation of Akt and Erk in response to EGF than MCF7 cells (Figure 6C). While EGF had minimal effects on IκB activation (Figure 6C), TNF activated IκB to similar levels in both cells but had minimal effects on Akt and Erk activation (Figure 6C). We also analyzed the activity of phosphatidylinositol-kinase-3-catalytic-alpha (PIK3CA) isoform after treatment with EGF and TNF in BT549 and MCF7 cells. BT549 cells treated with EGF (5 min) showed significant increase in PIK3CA activity compared to MCF7 cells (Supplementary Figure 5A). On the other hand, TNF showed no effect on PIK3CA activity in both cell lines (Supplementary Figure 5B).

### Akt activation is critical for regulating CXCL2 in TNBC BT549 cells

**Figure 7:**
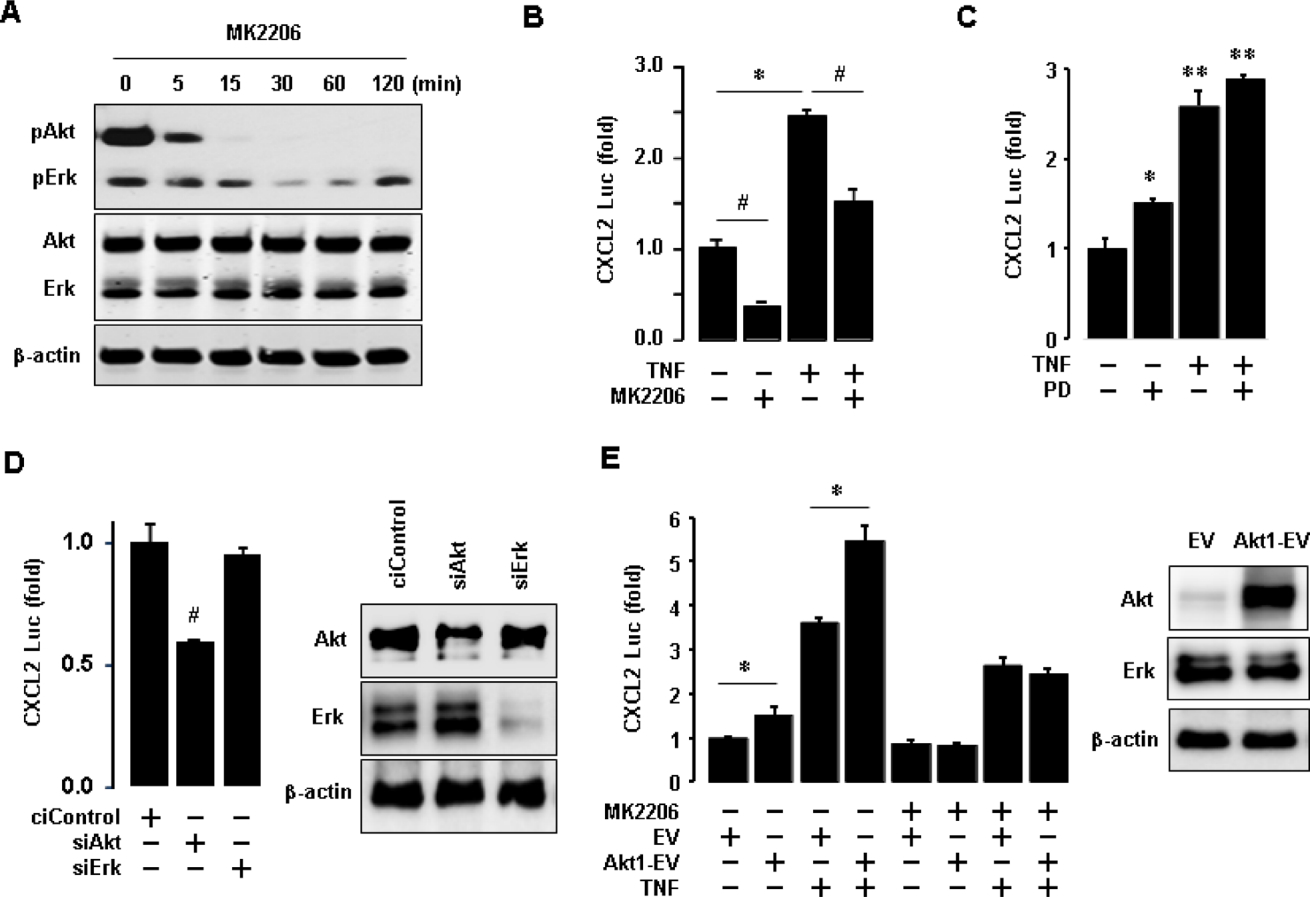
Involvement of Akt in regulating CXCL2 in BT549 cells (**A**) Effects of MK2206 (Akt inhibitor) on Akt activation in BT549 cells. β-actin was used as the loading control. (**B**) Effects of MK2206 on basal and TNF-induced levels of CXCL2 promoter activity. (**C**) Effects of PD98059 (PD, Erk inhibitor) on basal and TNF-induced levels of CXCL2 promoter activity. (**D**) Effects of Akt and Erk knockdown on CXCL2 promoter activity. Knockdown of Akt and Erk was confirmed by western blots, with β-actin as the loading control. (**E**) Effects of Akt1 overexpression on basal and TNF-induced levels of CXCL2 promoter activity. Overexpression of Akt1 was confirmed by western blots, with β-actin as the loading control. The asterisks (^*^,^**^) and hash (^#^) indicate a statistically significant increase and decrease (*p* ≤ 0.05) as determined by ANOVA and Tukey's pairwise comparison, respectively. The Student's *t*-test was also performed for values marked with a horizontal line. EV: empty expression vector, Akt1-EV: Akt1 expression vector.

Since TNBC cells expressed high protein levels of EGFR as well as phosphorylated Akt and Erk (Figures 4D and 6A), we assessed the role of Akt and Erk signaling in regulating proinflammatory chemokines in BT549 cells. We generated a luciferase promoter of CXCL2 as a dominant EGF-induced chemokine in BT549 cells (Figure 5). We inhibited Akt with MK2206 and activated NF-κB signaling with TNF. Activated NF-κB signaling induces the expression of proinflammatory chemokines [28]. MK2206 specifically abrogated the Akt phosphorylation in BT549 cells but had minimal effects on Erk phosphorylation (Figure 7A). MK2206 also reduced CXCL2 promoter activity at basal and TNF-induced levels (Figure 7B). On the other hand, PD98056 (Erk inhibitor) enhanced basal CXCL2 promoter activity and did not attenuate TNF-induced CXCL2 promoter activity (Figure 7C). We also used commercial siRNAs to knockdown Akt and Erk expression, respectively. The knockdown of Akt significantly reduced CXCL2 promoter activity, while the knockdown of Erk did not alter the activity (Figure 7D). Furthermore, overexpression of Akt1 increased CXCL2 promoter activity at both basal and TNF-induced levels (Figure 7E). MK2206 reduced the Akt1-enhanced effects on CXCL2 promoter activity (Figure 7E).

### TGFα is a dominant EGFR ligand in TNBC cells

**Figure 8:**
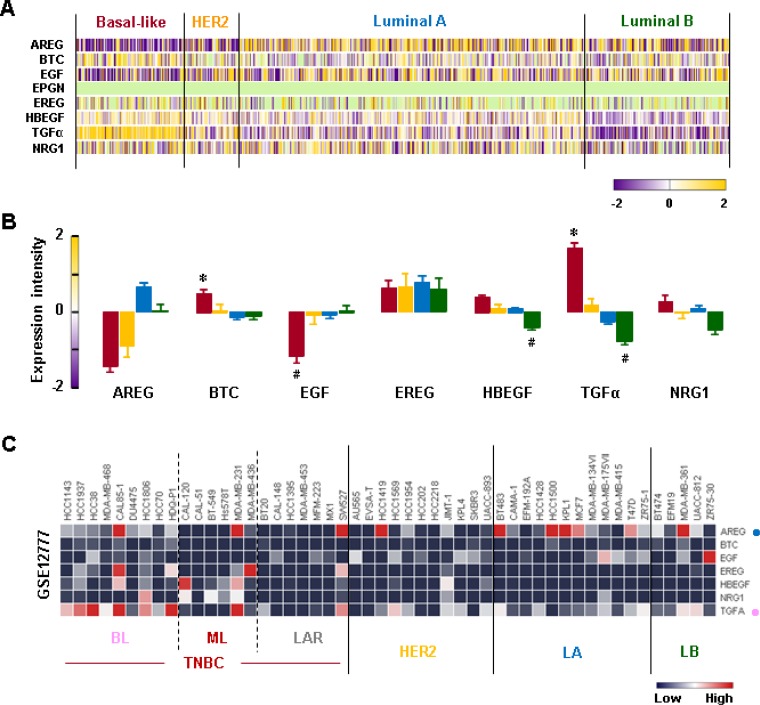
Expression profiles of EGFR-related ligands in BC tissues and cells (**A**) Heatmap for RNA expression levels of EGFR-related ligands in human BC tissues from TCGA-based dataset using Gitools 2.3.1. AREG: Amphiregulin, BTC: Betacellulin, EGF: epidermal growth factor, EREG: Epiregulin, HBEGF: Heparin-binding EGF-like growth factor, TGFα: Transforming growth factor alpha, NRG1: Neuregulin 1. (**B**) Statistical analysis for RNA expression levels of EGFR-related ligands in human BC tissues. The asterisk (^*^) and hash (^#^) indicate a statistically significant increase and decrease (*p* ≤ 0.05) as determined by ANOVA and Tukey's pairwise comparisons, respectively. (**C**) Heatmap for RNA expression levels of EGFR-related ligands based on analysis of the GEO dataset (Accession: GSE12777) for 51 human BC cell lines using Gitools 2.3.1. Pink and blue dots indicate dominant EGFR-related ligands in BL-TNBC and LA-BC cells, respectively.

We analyzed the gene expression profiles of the EGFR-related ligands in human BC tissues. We found that the BL-BC tissues exhibited higher levels of TGFα and betacellulin (BTC) than tissues from other BC subtypes (Figure 8A and 8B). Interestingly, the BL-BC tissues expressed the lowest levels of EGF compared to tissues from other BC subtypes (Figure 8A and 8B). Since enhanced TGFα and BTC levels in the BL-BC tissues could be due to tumor heterogeneity, we analyzed the NCBI GEO dataset on 51 human BC cell lines to exclude this possibility. Our analysis revealed that BL-TNBC cells expressed higher levels of TGFα than non-TNBC cell lines (Figure 8C and Supplementary Figure 6). We also found that amphiregulin (AREG) was a dominant EGFR ligand in LA-BC cells (Figure 8C and Supplementary Figure 6).

### Akt plays a role in regulating TGFα in TNBC BT549 cells

**Figure 9:**
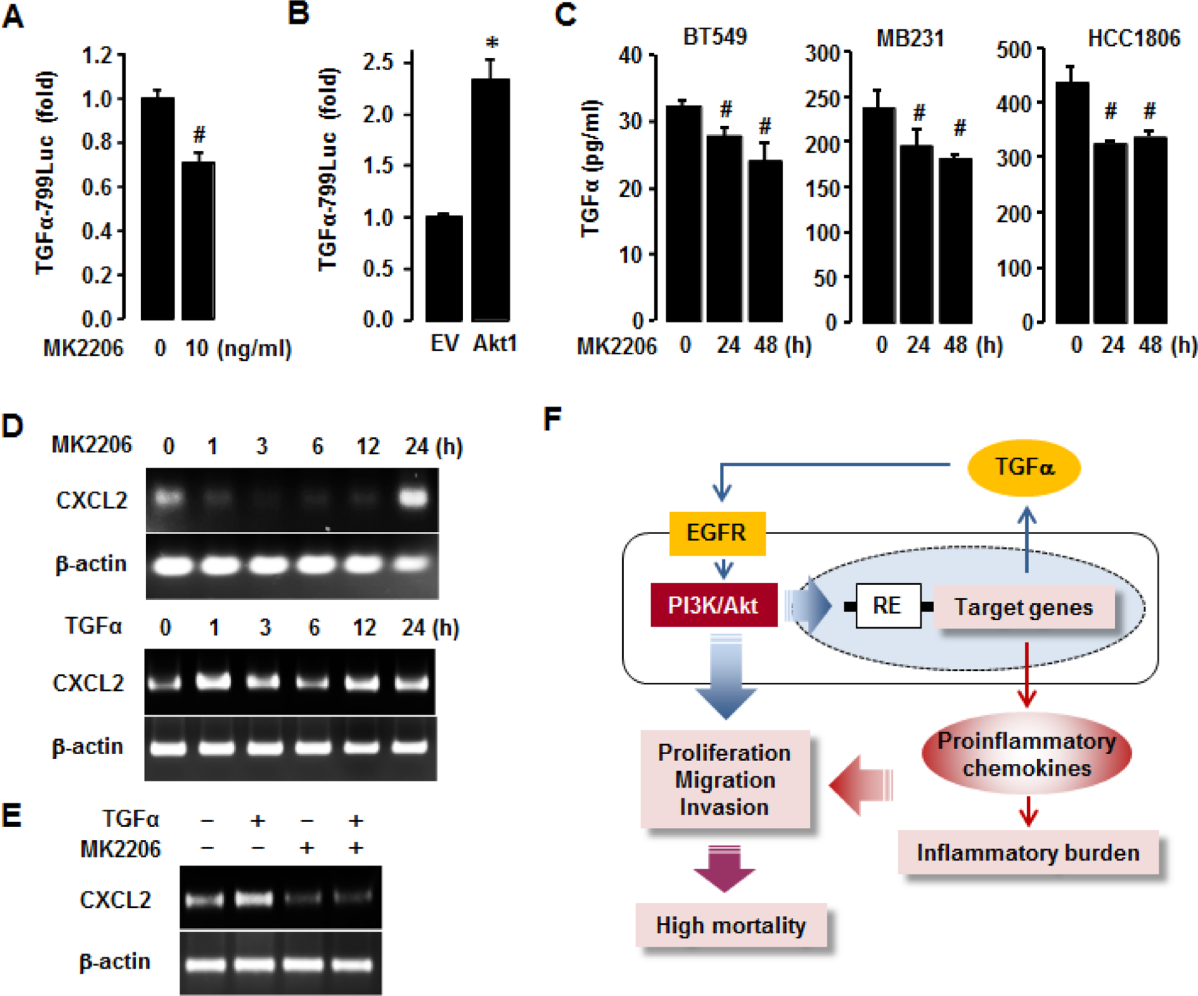
Involvement of Akt in regulating TGFα in TNBC cells (**A**) Effects of MK2206 (Akt inhibitor) on TGFα promoter activity in BT549 cells. (**B**) Enhanced effects of Akt1 overexpression on TGFα promoter activity in BT549 cells. (**C**) Effects of MK2206 on TGFα release from TNBC BT549, MB231 and HCC1806 cells. The asterisk (^*^) and hash (^#^) indicate a statistically significant increase and decrease (*p* ≤ 0.05) by the Student's *t*-test or ANOVA and Tukey's pairwise comparisons, respectively. (**D**) Effects of MK2206 and TGFα on CXCL2 mRNA expression levels in BT549 cells. (**E**) Combined treatment of TGFα and MK2206 on CXCL2 mRNA expression levels in BT549 cells. (**F**) Schematic representation of the molecular mechanisms that drive the enrichment of proinflammatory chemokines in TNBC cells. Blue arrows represent autocrine and paracrine TGFα-EGFR-Akt signaling, and red arrows represent inflammatory burden. Purple arrows represent the combined effect of Akt activation and proinflammatory chemokine enrichment. RE: responsive elements.

To delineate the role of Akt in regulating TGFα expression in TNBC BT549 cells, we performed TGFα luciferase promoter assay. MK2206 significantly decreased TGFα promoter activity in BT549 cells (Figure 9A), while overexpression of Akt1 increased its activity (Figure 9B). We further defined if Akt inhibition reduced TGFα release from TNBC cell lines (BT549, MB231, HCC1806) using an ELISA. MK2206 reduced TGFα release in these TNBC cells at 24 and 48 h post-treatment (Figure 9C). Additionally, we checked if Akt inhibition attenuated CXCL2 mRNA expression in TNBC BT549 cells. MK2206 downregulated CXCL2 mRNA expression as early as 1 h post-treatment, and the downregulation was reversed after 24 h post-treatment (Figure 9D). TGFα upregulated CXCL2 mRNA at various time points (Figure 9D). In addition, TGFα-induced CXCL2 mRNA expression level was abrogated by the pre-treatment of MK2206 (Figure 9E). MK2206 alone did not induce the expression level of CXCL2 mRNA (Figure 9E). These results indicate that the enhanced expression of proinflammatory chemokines in TNBC cells could be associated with the TGFα-EGFR-Akt axis via both autocrine and paracrine manner (Figure 9F).

## DISCUSSION

Our main findings highlight that TNBC cells express higher levels of proinflammatory chemokines such as CXCL1, 2, 3 and 8 than non-TNBC cells. TNBC cells also express higher levels of EGFR, exhibit more activation of Akt and a higher release of TGFα as the main EGFR ligand. The TGFα- and EGFR-mediated Akt activation may contribute to the enrichment of proinflammatory chemokines in TNBC cells. The aggressiveness of TNBC cells and poorer outcomes in patients with TNBC might be partly due to the enrichment of proinflammatory chemokines potentiated by the TGFα-EGFR-Akt signaling axis, which subsequently results in an increased inflammatory burden and produces a proinflammatory tumor microenvironment.

EGFR, a receptor tyrosine kinase of the HER family, is highly expressed in TNBC [29, 30]. Elevated EGFR expression level in tumors corresponds with aggressive basal-like phenotype and predicts for poor patient prognosis [31]. On the other hand, MCF7 and T47D (LA representative cell lines) could highly express ErbB3 and ErbB4, coinciding the case that ErbB3 level is paramount in mature luminal and luminal precursor cells and lowest in the basal cell of the breast [32]. Furthermore, LA subtypes highly express ErbB4 [33], being consistent with our analysis and results. Therefore, expression profiles of EGFR family indicate EGFR-driven in TNBC, HER2-driven in HER2, and ErbB3- and 4-driven in luminal subtypes. EGFR-targeted therapies could be an attractive option to treat TNBC, which lacks specific target genes. Unfortunately, the response rate of TNBC to EGFR inhibitors has only been 5% [34, 35]. To overcome this low efficacy, we would need to combine EGFR-targeted therapeutics with inhibitors for other targets. TNBC cells express high levels of TGFα, a ligand for EGFR. Therefore, it is possible that TGFα-mediated EGFR signaling promotes the aggressiveness of TNBC via both autocrine and paracrine signaling. Interestingly, TGFα is implicated in the growth and invasion of ovarian cancer cells via the activation of EGFR signaling [36]. Moreover, a significant association exists between TGFα expression and angiogenesis in invasive BC [37]. Overexpression of TGFα contributes to resistance to EGFR inhibitor cetuximab in colorectal cancer cells [38]. HER2-BC tumors respond poorly to lapatinib and capecitabine due to high serum TGFα levels [39]. TGFα is highly associated with axillary lymph node metastasis and low survival rates among BC patients [40]. Co-expression of EGFR and TGFα worsens the prognosis of patients with aggressive BC [41]. Therefore, it is possible that the TGFα-EGFR signaling axis promotes cancer cell proliferation, invasion, and metastasis, all of which drive the aggressiveness of TNBC and lead to poor response to EGFR inhibitors. Usually, overexpression of growth factor receptors like EGFR in human tumors may contribute to Akt activation [42]. Our findings are consistent with this observation in that TNBC cells with higher levels of EGFR expression exhibited more activation of Akt and Erk than non-TNBC cells. In addition, exposure to EGF, a main ligand for EGFR, led to induce activity of PIK3CA in TNBC cells compared to non-TNBC cells. Cumulative evidence indicated a high frequency of mutations in the *PIK3CA* gene, which codes for p110α in various human cancers [43, 44]. The mutations in PIK3CA lead to induce PI3K kinase activity followed by Akt activation, leading to increased cell survival and cell cycle progression [45]. Moreover, the exposure to EGF induces the higher levels of phosphorylated Akt and Erk in TNBC cells compared to non-TNBC cells. EGFR-activated two major pathways in BC are the PI3K/Akt and MAPK/Erk signaling pathways [46] and TNF-activated major pathway is NF-kB signaling. EGF had little effect on NF-kB signaling pathway (Figure 6C), but more effects on the PI3K/Akt and MAPK/Erk. The relationship between EGFR activation and NF-κB signaling is very controversial in other model systems. EGF treatment contributed to NF-κB activity in human proximal tubule cells and in pancreatic cancer [47, 48]. In contrast, EGF did not activate NF-κB or alter NF-κB activation by TNF in chondrocytes [49]. Even ovarian cancer cells appeared induction of proinflammatory chemokines in EGF- or TNF-responsive manner [28]. Despite little activation of NF-κB, EGF is likely to broadly induce CCL20, CXCL1-3 and CXCL8 through Akt activation in BT549 cell (Figure 5). Therefore, EGF-activated Akt looks like potentiate these chemokines rather than NF-κB, driving us to focus on Akt activation to induce these chemokines. Activated Akt and Erk pathways are potential prognostic markers for TNBC [50] and phosphorylation of Akt appears to be significantly higher in TNBC samples [51]. These reports indicate that EGFR-mediated signaling is more active in TNBC cells at both basal and induced levels than in non-TNBC cells. This enhancement in EGFR-mediated signaling likely promotes the aggressiveness of TNBC. Akt signaling plays a crucial role in the initiation and progression of breast tumorigenesis because it regulates various cellular processes such as proliferation, survival, and metabolism [52, 53]. Human tumors frequently exhibit alterations in Akt, which may contribute to cancer development and/or progression [54, 55]. Interestingly, Akt activation may also play a role in regulating TGFα expression as a main ligand for EGFR in TNBC. In this regard, we found that overexpression of Akt induced TGFα promoter activity, while Akt inhibitor MK2206 downregulated the activity. PI3K/Akt inhibition has been shown to reduce dexamethasone-induced TGFα promoter activity and mRNA levels in astrocytes [56]. In agreement with this previous observation, we found that MK2206 reduced TGFα production in and release from TNBC cells. Based on previously published results and our new findings, we conclude that the Akt pathway likely plays a critical role in regulating TGFα production in TNBC cells. Therefore, overexpression of EGFR and constitutive release of TGFα from TNBC can enhance the potency of Akt activation, subsequently potentiating the TGFα-EGFR signaling axis in both autocrine and paracrine manner (Figure 9F, blue lines).

In this study, we observed that TNBC cells expressed high levels of proinflammatory chemokines CXCL1 and 8. Several previous studies have implicated the role of proinflammatory chemokines in BC progression and metastasis. For instance, CXCR2 ligands such as CXCL1, 2, 3, 5, 6 and 8 are associated with angiogenesis [24, 57]. BC cells that overexpress CXCL1 and 2 are primed for survival at metastatic sites, which links cancer chemoresistance [58]. TNF-activated mesenchymal stromal cells express CXCR2 ligands including CXCL1, 2 and 5 and promote BC metastasis [59]. Elevated CXCL1 expression in BC stroma correlates positively with tumor grade, disease recurrence, and decreased patient survival [60]. Downregulation of CXCL1 and 2 inhibits BC metastasis [61]. Concurrent inhibition of IL-6 and CXCL8 expression in TNBC cells inhibits colony formation, cell survival, and tumor growth [62]. Targeting CXCR2 enhances the antitumor activity of paclitaxel and inhibits mammary tumor growth, angiogenesis, and lung metastasis [63]. These findings indicate that proinflammatory chemokines play a critical role in TNBC progression and metastasis by forming an inflammatory tumor microenvironment that can lead to chemoresistance. The AR and GATA3 were enriched in LAR-TNBC subtype and were absent in ML-TNBC subtype [15]. A high level of “luminal-like” genes such as AR and GATA3 in LAR-TNBC subtype has a relatively favorable prognosis compared to tumors expressing cancer stem cell markers [15]. GATA3 is recognized as a marker of luminal ER-positive breast tumor [15, 64, 65]. Therefore, it is noteworthy to mention that BL- and ML-TNBC subtypes highly express CXCL1, 2, 5, and 8 compared to LAR-TNBC with luminal-like characteristics. On the other hand, non-TNBC cells such as T47D highly express CXCL10. Cumulative evidence suggested that increased expression levels of CXCL9 and CXCL10 induced tumor-infiltrating CD8+T cells, leading to reduced cancer progression or metastasis and enhanced survival in ovarian and colon cancer patients [66–72]. Of note, the infiltration of CD8^+^ T cells in the tumor microenvironment had been suggested to have antitumor effects [72]. In addition, CXCL10 has angiostatic role, blocking microvascular endothelial cell migration and proliferation [23, 24]. Therefore, overexpression of CXCL10 indicates attenuation of BC aggressiveness.

Previously, we demonstrated that EGF or TNF could induce the expression of proinflammatory chemokines such as CXCL1, 2, 3 and 8 in ovarian cancer cells via NF-κB, Akt and Erk signaling pathways [28]. Here, we used the CXCL2 promoter to confirm the involvement of the TGFα-EGFR-Akt signaling axis in the enrichment of proinflammatory chemokines in TNBC cells. Our promoter, knockdown, inhibitor, and overexpression experiments revealed that Akt activation regulated CXCL2 expression. However, Erk activation did not upregulate CXCL2 expression in TNBC cells. In addition, TGFα upregulated CXCL2 mRNA expression levels in TNBC cells. A previous study showed that EGF could enhance CXCL5 expression in endothelial cells via EGFR signaling [73]. In bronchial epithelial cells, 1-nitropyrene/1-aminopyrene and airborne particulates induce CXCL8 expression via the TGFα-EGFR signaling pathway [74, 75]. EGFR activation is essential for CXCL8 release from intestinal epithelial cells in response to challenge by *Escherichia coli* O157:H7 flagellin [76]. All these previous reports support our current observation that CXCL1 and 8 are the dominant chemokines in TNBC cells which express high levels of EGFR. In addition, CXCL8 can stimulate cell proliferation in non-small cell lung cancer through EGFR transactivation [77]. CXCR2 ligands like CXCL1 and 2 are closely associated with EGFR transactivation [27, 78]. We postulate that CXCL1 and 8 potentiate EGFR transactivation in TNBC cells to enhance the cancer aggressiveness and chemoresistance. Targeting both CXCL8 and EGFR with antibodies inhibits metastasis of human BC xenografts [79]. Based on previously published results and our current findings, we conclude that the TGFα-EGFR-Akt signaling drives the enrichment of proinflammatory chemokines in TNBC cells. On the other hand, non-TNBC cells exhibit weak activation of TGFα-EGFR-Akt signaling, resulting in low expression levels of proinflammatory chemokines and a low inflammatory burden.

Contrary to non-TNBC cells, TNBC cells express high levels of EGFR and TGFα, which can activate Akt signaling via the TGFα-EGFR axis. This activation promotes cell proliferation, migration, and invasion (Figure 9F, blue lines), all of which are critical for cancer progression. Moreover, the TGFα-EGFR-Akt signaling axis in TNBC cells can be potentiated in both autocrine and paracrine manner, resulting in the enrichment of proinflammatory chemokines. The higher levels of proinflammatory chemokines subsequently generate a higher inflammatory burden, thereby accelerating TNBC progression and metastasis (Figure 9F). The aggressiveness of TNBC ultimately results in higher mortality rates among BC patients.

## MATERIALS AND METHODS

### Reagents

Recombinant human proteins and inhibitors were purchased as follows: TNF and EGF from Cell Signaling Technology (Beverly, MA, USA), TGFα from Raybiotech Inc. (Norcross, GA, USA), and MK2206 (Akt inhibitor) and PD98059 (Erk inhibitor) from Cayman Chemical Company (Ann Arbor, MI, USA). Antibodies were purchased as follows: NF-κB family members, IκB, EGFR, Erk1/2, Akt, and their phosphorylated forms such as pIκB (Ser32/36), pEGFR (Tyr1173), pErk1/2 (Thr202/Tyr204) and pAkt (Ser473) from Cell Signaling Technology (Beverly, MA, USA); and ErbB isoforms, p65, and β-actin from Santa Cruz Biotechnology (Santa Cruz, CA, USA). A customized PCR array for chemokines, SYBR^®^ Green Master Mix and RNeasy Mini Kit came from SABiosciences in Qiagen (Frederick, MD, USA). TGFα ELISA kits were from Raybiotech Inc. (Norcross, GA, USA). Chemiluminescent detection kits were from GE Healthcare (Piscataway, NJ, USA). Antisense and sense oligonucleotides were obtained from Eurofins MWG Operon (Huntsville, AL, USA). Lipofectamine 2000 and all liquid culture media were acquired from Invitrogen (Grand Island, NY, USA). The siRNAs for control, Akt and Erk were purchased from Cell Signaling Technology (Beverly, MA, USA). The Luciferase Reporter Assay System was obtained from Promega (Madison, WI, USA).

### Cell culture

Human TNBC (MB468, MB231, BT549 and HCC1806) and non-TNBC (MCF7, T47D) cell lines were purchased from the American Type Culture Collection (Manassas, VA, USA). The cells were cultured in RPMI medium containing penicillin/streptomycin and 10% fetal bovine serum (FBS) at 37° C in a water-saturated atmosphere of 95% air and 5% CO_2_. After an overnight culture in 24- and/or 6-well plates to allow cellular attachment, the medium was removed and fresh medium without FBS was added to eliminate the effects of serum, per se. Where indicated, the cells were treated with vehicle (phosphate-buffered saline, PBS), 10 ng/ml EGF, 10 ng/ml TNF, and incubations continued for the indicated time periods. Treatments with reagents were described in detail in the Results.

### Western blot

Whole-cell lysates were prepared, fractionated on SDS-polyacrylamide gels, and transferred to nitrocellulose membranes according to established procedures [80]. The following primary antibodies were used: EGFR, Akt, Erk, IκB, and their phosphorylated forms (Cell Signaling Technology, Beverly, MA). The protein bands were visualized by chemiluminescence detection kits. β-actin was used as the loading control.

### RT-PCR

Total RNA was isolated using RNeasy Mini Kit (Qiagen, Frederick, MD, USA) according to manufacturer's instructions. The reverse transcriptase reaction conditions using random primers began at 42° C for 60 min, followed by 94° C for 10 min. Specific primers were designed as follows: 5′- GCA GGG AAT TCA CCT CAA G-3′ (sense) and 5′- GGG GTT GAG ACA AGC TTT C -3′ (antisense) for CXCL2 and 5′- CCT CAT GAA GAT CCT CAC CG -3′ (sense) and 5′- CCA TCT CTT GCT CGA AGT CC-3′ (antisense) for β-actin. β-actin was used as an internal control. PCR was performed under the following conditions: denaturation at 94 C for 1 min, annealing at 60 C for 1 min, and extension at 74 C for 1 min with 30 cycles. Amplified PCR products were analyzed by electrophoresis on 2% agarose gels and fluorescent images were photographed under UV light.

### Chemokine PCR array

After isolating total RNA from cancer cells and eliminating genomic DNA, the RT reaction was performed at 42° C for 15 min, followed by 94° C for 5 min. A real-time PCR reaction for chemokines was performed according to manufacturer's instructions using a Bio-Rad CFX96 instrument (Hercules, CA, USA) and the following two-step cycling program: one cycle at 95° C for 10 min, and 40 cycles at 95° C for 15 sec and at 60° C for 1 min. Data analysis was performed using a web-based PCR array data analysis software (http://saweb2.sabiosciences.com/pcr/arrayanalysis.php) provided by SABiosciences in Qiagen (Frederick, MD, USA).

### Construction of the CXCL2 and TGFα promoters

Human TGFα promoter was generated as described previously [81]. The DNA fragment of the human CXCL2 was generated by PCR using genomic DNA isolated from BT549 cells. The following primers were designed: 5′-AGA GAA GTA ACT CCC CCC GG-3′ (sense) containing an *XhoI* site and 5′-GCT CTG TGG CTC TCC GAG AA-3′ (antisense) containing a *HindIII* site at −379 and +10 bp position of CXCL2 promoter, respectively. The PCR reaction was performed with 35 cycles at 94° C for 30 sec, 58° C for 30 sec, and 74° C for 1 min, followed by a final extension at 74° C for 10 min. The amplified CXCL2 DNA fragment was digested with *XhoI* and *Hind III*, and purified according to manufacturer ‘s instructions (Gel Extraction System, Qiagen, Valencia, CA). The purified CXCL2 promoter fragment was subcloned into the *XhoI* and *HindIII* sites in the pGL4.12-basic vector (Promega, Madison, WI, USA). The CXCL2 promoter-luciferase construct was confirmed by DNA sequencing.

### Transient transfection and luciferase assay

CXCL2 and TGFα promoter activities were assessed using generated CXCL2-379LUC and TGFα-799LUC vectors. BT549 cells at approximately 50% confluency in 24-well plates were washed once with fresh media without additives, transiently transfected with the target plasmids using Lipofectamine 2000 (Invitrogen, Grand Island, NY, USA), and incubated for 24 h at 37° C. The transfected cells were treated as outlined in Results. The cells were then rinsed with cold PBS and lysis buffer (Promega, Madison, WI, USA) was added. The cell lysates were used for luciferase activity determination using a microplate luminometer. Luciferase activity, expressed as relative light units, was normalized to measured protein levels.

### Enzyme-linked immunosorbent assay (ELISA)

The levels of secreted TGFα from representative TNBC cell lines (BT549, MB231 and HCC1806) in their basal or post-MK2206 treatment (outlined in Results) were assessed by an ELISA kit (Raybiotech Inc., Norcross, GA, USA) according to the manufacturer's instructions. The PIK3CA activity in BT549 and MCF7 cells (lysate) after time-dependent treatment with EGF and TNF was analyzed by an ELISA kit (Aviva Systems Biology Corporation, San Diego, CA, USA) according to the manufacturer's instructions. The optical density of each well was determined at 450 nm with a microplate reader. Assays were calibrated using serial dilutions of recombinant human TGFα and lyophilized standard of PIK3CA, respectively, according to the manufacturer's instructions.

### Data analysis from GEO and the TCGA datasets

Data analysis was performed using the NCBI GEO microarray dataset (http://www.ncbi.nlm.nih.gov/geo/) with accession number GSE12777. Raw microarray data for chemokines were obtained from RNA expression levels in 51 human BC cell lines. The basal chemokine expression levels were determined by global gene expression profiling of BC cell lines, while molecular subtyping was determined using gene expression and HER2 status by fluorescent *in situ* hybridization. We employed Gitools 2.3.1 (http://www.gitools.org), an open-source tool based on Oracle Java 7, to analyze and visualize the genomic data via interactive heat-maps [82]. The breast invasive carcinoma dataset from TCGA individual projects was used to analyze different BC subtypes (http://www.gitools.org/datasets/tcga).

### Transfection of BC cells with Erk and Akt siRNAs

BT549 cells at approximately 50% confluency in six-well plates were washed once with 1% FBS fresh media without additives. Then cells were transiently transfected with Control, Akt and Erk siRNAs (final concentration: 100 nM) for 48 h at 37° C using Lipofectamine solution. Knockdown was confirmed by western blot and the cells were treated as outlined in Results.

### Statistical analysis

Data were analyzed using the paired Student's *t*-test and one-way analysis of variance (ANOVA) as appropriate. If statistical significance (*p* ≤ 0.05) was determined by ANOVA, the data were further analyzed by Tukey's pairwise comparisons to detect specific differences between treatments

## SUPPLEMENTARY MATERIALS FIGURES



## Abbreviations

ANOVA: one-way analysis of variance
AREG: amphiregulin
BC: breast cancer
BL1: basal-like 1
BL2: basal-like 2
BTC: betacellulin
EGF: epidermal growth factor
EGFR: epidermal growth factor receptor
ELISA: enzyme-linked immunosorbent assay
EMT: epithelial-to-mesenchymal transition
ER: estrogen receptor
EREG: epiregulin
FBS: fetal bovine serum
GEO: gene expression omnibus
HER-2: epidermal growth factor receptor 2
HBEGF: heparin-binding EGF-like growth factor
LA: luminal A
LAR: luminal androgen
LB: luminal B
ML: mesenchymal-like
NCBI: National Center for Biotechnology Information
NRG1: neuregulin 1
PBS: phosphate buffered saline
PIK3CA: phosphatidylinositol-kinase-3-catalytic-alpha
PR: progesterone receptor
TCGA: The Cancer genome Atlas
TGFα: transforming growth factor alpha
TNBC: triple-negative breast cancer
TNF: tumor necrosis factor alpha.

## References

[1] Siegel RL, Miller KD, Jemal A (2017). Cancer Statistics, 2017. CA Cancer J Clin.

[2] Perou CM, Sørlie T, Eisen MB, van de Rijn M, Jeffrey SS, Rees CA, Pollack JR, Ross DT, Johnsen H, Akslen LA, Fluge O, Pergamenschikov A, Williams C (2000). Molecular portraits of human breast tumours. Nature.

[3] Sorlie T, Tibshirani R, Parker J, Hastie T, Marron JS, Nobel A, Deng S, Johnsen H, Pesich R, Geisler S, Demeter J, Perou CM, Lønning PE (2003). Repeated observation of breast tumor subtypes in independent gene expression data sets. Proc Natl Acad Sci USA.

[4] Goldhirsch A, Wood WC, Coates AS, Gelber RD, Thürlimann B, Senn HJ, and Panel members (2011). Strategies for subtypes—dealing with the diversity of breast cancer: highlights of the St. Gallen International Expert Consensus on the Primary Therapy of Early Breast Cancer 2011. Ann Oncol.

[5] Hon JD, Singh B, Sahin A, Du G, Wang J, Wang VY, Deng FM, Zhang DY, Monaco ME, Lee P (2016). Breast cancer molecular subtypes: from TNBC to QNBC. Am J Cancer Res.

[6] Kalimutho M, Parsons K, Mittal D, López JA, Srihari S, Khanna KK (2015). Targeted Therapies for Triple-Negative Breast Cancer: Combating a Stubborn Disease. Trends Pharmacol Sci.

[7] Haffty BG, Yang Q, Reiss M, Kearney T, Higgins SA, Weidhaas J, Harris L, Hait W, Toppmeyer D (2006). Locoregional relapse and distant metastasis in conservatively managed triple negative early-stage breast cancer. J Clin Oncol.

[8] Anders CK, Carey LA (2009). Biology, metastatic patterns, and treatment of patients with triple-negative breast cancer. Clin Breast Cancer.

[9] Cadoo KA, Fornier MN, Morris PG (2013). Biological subtypes of breast cancer: current concepts and implications for recurrence patterns. Q J Nucl Med Mol Imaging.

[10] Dent R, Trudeau M, Pritchard KI, Hanna WM, Kahn HK, Sawka CA, Lickley LA, Rawlinson E, Sun P, Narod SA (2007). Triple-negative breast cancer: clinical features and patterns of recurrence. Clin Cancer Res.

[11] Lehmann BD, Bauer JA, Chen X, Sanders ME, Chakravarthy AB, Shyr Y, Pietenpol JA (2011). Identification of human triple-negative breast cancer subtypes and preclinical models for selection of targeted therapies. J Clin Invest.

[12] Chen X, Li J, Gray WH, Lehmann BD, Bauer JA, Shyr Y, Pietenpol JA (2012). TNBCtype: A Subtyping Tool for Triple-Negative Breast Cancer. Cancer Inform.

[13] Burstein MD, Tsimelzon A, Poage GM, Covington KR, Contreras A, Fuqua SA, Savage MI, Osborne CK, Hilsenbeck SG, Chang JC, Mills GB, Lau CC, Brown PH (2015). Comprehensive genomic analysis identifies novel subtypes and targets of triple-negative breast cancer. Clin Cancer Res.

[14] Lehmann BD, Jovanović B, Chen X, Estrada MV, Johnson KN, Shyr Y, Moses HL, Sanders ME, Pietenpol JA (2016). Refinement of Triple-Negative Breast Cancer Molecular Subtypes: Implications for Neoadjuvant Chemotherapy Selection. PLoS One.

[15] Yu KD, Zhu R, Zhan M, Rodriguez AA, Yang W, Wong S, Makris A, Lehmann BD, Chen X, Mayer I, Pietenpol JA, Shao ZM, Symmans WF, Chang JC (2013). Identification of prognosis-relevant subgroups in patients with chemoresistant triple-negative breast cancer. Clin Cancer Res.

[16] Wathieu H, Issa NT, Fernandez AI, Mohandoss M, Tiek DM, Franke JL, Byers SW, Riggins RB, Dakshanamurthy S (2017). Differential prioritization of therapies to subtypes of triple negative breast cancer using a systems medicine method. Oncotarget.

[17] Ali S, Lazennec G (2007). Chemokines: novel targets for breast cancer metastasis. Cancer Metastasis Rev.

[18] Butcher EC, Williams M, Youngman K, Rott L, Briskin M (1999). Lymphocyte trafficking and regional immunity. Adv Immunol.

[19] Zlotnik A, Yoshie O (2000). Chemokines: a new classification system and their role in immunity. Immunity.

[20] Campbell JJ, Butcher EC (2000). Chemokines in tissue-specific and microenvironment-specific lymphocyte homing. Curr Opin Immunol.

[21] Haghnegahdar H, Du J, Wang D, Strieter RM, Burdick MD, Nanney LB, Cardwell N, Luan J, Shattuck-Brandt R, Richmond A (2000). The tumorigenic and angiogenic effects of MGSA/GRO proteins in melanoma. J Leukoc Biol.

[22] Rubie C, Frick VO, Wagner M, Schuld J, Gräber S, Brittner B, Bohle RM, Schilling MK (2008). ELR+ CXC chemokine expression in benign and malignant colorectal conditions. BMC Cancer.

[23] Palacios-Arreola MI, Nava-Castro KE, Castro JI, García-Zepeda E, Carrero JC, Morales-Montor J (2014). The role of chemokines in breast cancer pathology and its possible use as therapeutic targets. J Immunol Res.

[24] Strieter RM, Burdick MD, Mestas J, Gomperts B, Keane MP, Belperio JA (2006). Cancer CXC chemokine networks and tumour angiogenesis. Eur J Cancer.

[25] Richmond A (2002). Nf-kappa B, chemokine gene transcription and tumour growth. Nat Rev Immunol.

[26] Sarvaiya PJ, Guo D, Ulasov I, Gabikian P, Lesniak MS (2013). Chemokines in tumor progression and metastasis. Oncotarget.

[27] Dong YL, Kabir SM, Lee ES, Son DS (2013). CXCR2-driven ovarian cancer progression involves upregulation of proinflammatory chemokines by potentiating NF-κB activation via EGFR-transactivated Akt signaling. PLoS One.

[28] Son DS, Kabir SM, Dong Y, Lee E, Adunyah SE (2013). Characteristics of chemokine signatures elicited by EGF and TNF in ovarian cancer cells. J Inflamm (Lond).

[29] Sarrió D, Rodriguez-Pinilla SM, Hardisson D, Cano A, Moreno-Bueno G, Palacios J (2008). Epithelial-mesenchymal transition in breast cancer relates to the basal-like phenotype. Cancer Res.

[30] Gluz O, Liedtke C, Gottschalk N, Pusztai L, Nitz U, Harbeck N (2009). Triple-negative breast cancer—current status and future directions. Ann Oncol.

[31] Ali R, Wendt MK (2017). The paradoxical functions of EGFR during breast cancer progression. Signal Transduct Target Ther.

[32] Balko JM, Miller TW, Morrison MM, Hutchinson K, Young C, Rinehart C, Sánchez V, Jee D, Polyak K, Prat A, Perou CM, Arteaga CL, Cook RS (2012). The receptor tyrosine kinase ErbB3 maintains the balance between luminal and basal breast epithelium. Proc Natl Acad Sci USA.

[33] Hoadley KA, Weigman VJ, Fan C, Sawyer LR, He X, Troester MA, Sartor CI, Rieger-House T, Bernard PS, Carey LA, Perou CM (2007). EGFR associated expression profiles vary with breast tumor subtype. BMC Genomics.

[34] Ueno NT, Zhang D (2011). Targeting EGFR in Triple Negative Breast Cancer. J Cancer.

[35] Nakai K, Hung MC, Yamaguchi H (2016). A perspective on anti-EGFR therapies targeting triple-negative breast cancer. Am J Cancer Res.

[36] Lau TS, Chan LK, Wong EC, Hui CW, Sneddon K, Cheung TH, Yim SF, Lee JH, Yeung CS, Chung TK, Kwong J (2017). A loop of cancer-stroma-cancer interaction promotes peritoneal metastasis of ovarian cancer via TNFα-TGFα-EGFR. Oncogene.

[37] Soares R, Pereira MB, Silva C, Amendoeira I, Wagner R, Ferro J, Schmitt FC (2000). Expression of TGF-alpha and EGFR in Breast Cancer and its Relation to Angiogenesis. Breast J.

[38] Troiani T, Martinelli E, Napolitano S, Vitagliano D, Ciuffreda LP, Costantino S, Morgillo F, Capasso A, Sforza V, Nappi A, De Palma R, D’Aiuto E, Berrino L (2013). Increased TGF-α as a mechanism of acquired resistance to the anti-EGFR inhibitor cetuximab through EGFR-MET interaction and activation of MET signaling in colon cancer cells. Clin Cancer Res.

[39] Rhee J, Han SW, Cha Y, Ham HS, Kim HP, Oh DY, Im SA, Park JW, Ro J, Lee KS, Park IH, Im YH, Bang YJ, Kim TY (2011). High serum TGF-α predicts poor response to lapatinib and capecitabine in HER2-positive breast cancer. Breast Cancer Res Treat.

[40] Auvinen PK, Lipponen PK, Kataja VV, Johansson RT, Syrjänen KJ (1996). Prognostic significance of TGF-alpha expression in breast cancer. Acta Oncol.

[41] Umekita Y, Ohi Y, Sagara Y, Yoshida H (2000). Co-expression of epidermal growth factor receptor and transforming growth factor-alpha predicts worse prognosis in breast-cancer patients. Int J Cancer.

[42] Schlegel J, Piontek G, Mennel HD (2002). Activation of the anti-apoptotic Akt/protein kinase B pathway in human malignant gliomas *in vivo*. Anticancer Res.

[43] Samuels Y, Wang Z, Bardelli A, Silliman N, Ptak J, Szabo S, Yan H, Gazdar A, Powell SM, Riggins GJ, Willson JK, Markowitz S, Kinzler KW (2004). High frequency of mutations of the PIK3CA gene in human cancers. Science.

[44] Levine DA, Bogomolniy F, Yee CJ, Lash A, Barakat RR, Borgen PI, Boyd J (2005). Frequent mutation of the PIK3CA gene in ovarian and breast cancers. Clin Cancer Res.

[45] Huang CH, Mandelker D, Gabelli SB, Amzel LM (2008). Insights into the oncogenic effects of PIK3CA mutations from the structure of p110alpha/p85alpha. Cell Cycle.

[46] Liu Q, Yu S, Zhao W, Qin S, Chu Q, Wu K (2018). EGFR-TKIs resistance via EGFR-independent signaling pathways. Mol Cancer.

[47] Häussler U, von Wichert G, Schmid RM, Keller F, Schneider G (2005). Epidermal growth factor activates nuclear factor-kappaB in human proximal tubule cells. Am J Physiol Renal Physiol.

[48] Liptay S, Weber CK, Ludwig L, Wagner M, Adler G, Schmid RM (2003). Mitogenic and antiapoptotic role of constitutive NF-kappaB/Rel activity in pancreatic cancer. Int J Cancer.

[49] Klooster AR, Bernier SM (2005). Tumor necrosis factor alpha and epidermal growth factor act additively to inhibit matrix gene expression by chondrocyte. Arthritis Res Ther.

[50] Hashimoto K, Tsuda H, Koizumi F, Shimizu C, Yonemori K, Ando M, Kodaira M, Yunokawa M, Fujiwara Y, Tamura K (2014). Activated PI3K/AKT and MAPK pathways are potential good prognostic markers in node-positive, triple-negative breast cancer. Ann Oncol.

[51] Umemura S, Yoshida S, Ohta Y, Naito K, Osamura RY, Tokuda Y (2007). Increased phosphorylation of Akt in triple-negative breast cancers. Cancer Sci.

[52] Bellacosa A, Kumar CC, Di Cristofano A, Testa JR (2005). Activation of AKT kinases in cancer: implications for therapeutic targeting. Adv Cancer Res.

[53] Dey N, De P, Leyland-Jones B (2017). PI3K-AKT-mTOR inhibitors in breast cancers: from tumor cell signaling to clinical trials. Pharmacol Ther.

[54] Altomare DA, Testa JR (2005). Perturbations of the AKT signaling pathway in human cancer. Oncogene.

[55] Mitsiades CS, Mitsiades N, Koutsilieris M (2004). The Akt pathway: molecular targets for anti-cancer drug development. Curr Cancer Drug Targets.

[56] Karki P, Johnson J, Son DS, Aschner M, Lee E (2017). Transcriptional Regulation of Human Transforming Growth Factor-α in Astrocytes. Mol Neurobiol.

[57] Keane MP, Burdick MD, Xue YY, Lutz M, Belperio JA, Strieter RM (2004). The chemokine receptor, CXCR2, mediates the tumorigenic effects of ELR+ CXC chemokines. Chest.

[58] Acharyya S, Oskarsson T, Vanharanta S, Malladi S, Kim J, Morris PG, Manova-Todorova K, Leversha M, Hogg N, Seshan VE, Norton L, Brogi E, Massagué J (2012). A CXCL1 paracrine network links cancer chemoresistance and metastasis. Cell.

[59] Yu PF, Huang Y, Han YY, Lin LY, Sun WH, Rabson AB, Wang Y, Shi YF (2017). TNFα-activated mesenchymal stromal cells promote breast cancer metastasis by recruiting CXCR2+ neutrophils. Oncogene.

[60] Zou A, Lambert D, Yeh H, Yasukawa K, Behbod F, Fan F, Cheng N (2014). Elevated CXCL1 expression in breast cancer stroma predicts poor prognosis and is inversely associated with expression of TGF-β signaling proteins. BMC Cancer.

[61] Kronski E, Fiori ME, Barbieri O, Astigiano S, Mirisola V, Killian PH, Bruno A, Pagani A, Rovera F, Pfeffer U, Sommerhoff CP, Noonan DM, Nerlich AG (2014). miR181b is induced by the chemopreventive polyphenol curcumin and inhibits breast cancer metastasis via down-regulation of the inflammatory cytokines CXCL1 and -2. Mol Oncol.

[62] Hartman ZC, Poage GM, den Hollander P, Tsimelzon A, Hill J, Panupinthu N, Zhang Y, Mazumdar A, Hilsenbeck SG, Mills GB, Brown PH (2013). Growth of triple-negative breast cancer cells relies upon coordinate autocrine expression of the proinflammatory cytokines IL-6 and IL-8. Cancer Res.

[63] Sharma B, Nawandar DM, Nannuru KC, Varney ML, Singh RK (2013). Targeting CXCR2 enhances chemotherapeutic response, inhibits mammary tumor growth, angiogenesis, and lung metastasis. Mol Cancer Ther.

[64] Sørlie T, Perou CM, Tibshirani R, Aas T, Geisler S, Johnsen H, Hastie T, Eisen MB, van de Rijn M, Jeffrey SS, Thorsen T, Quist H, Matese JC (2001). Gene expression patterns of breast carcinomas distinguish tumor subclasses with clinical implications. Proc Natl Acad Sci USA.

[65] Voduc D, Cheang M, Nielsen T (2008). GATA-3 expression in breast cancer has a strong association with estrogen receptor but lacks independent prognostic value. Cancer Epidemiol Biomarkers Prev.

[66] Zhang L, Conejo-Garcia JR, Katsaros D, Gimotty PA, Massobrio M, Regnani G, Makrigiannakis A, Gray H, Schlienger K, Liebman MN, Rubin SC, Coukos G (2003). Intratumoral T cells, recurrence, and survival in epithelial ovarian cancer. N Engl J Med.

[67] Pagès F, Berger A, Camus M, Sanchez-Cabo F, Costes A, Molidor R, Mlecnik B, Kirilovsky A, Nilsson M, Damotte D, Meatchi T, Bruneval P, Cugnenc PH (2005). Effector memory T cells, early metastasis, and survival in colorectal cancer. N Engl J Med.

[68] Sato E, Olson SH, Ahn J, Bundy B, Nishikawa H, Qian F, Jungbluth AA, Frosina D, Gnjatic S, Ambrosone C, Kepner J, Odunsi T, Ritter G (2005). Intraepithelial CD8+ tumor-infiltrating lymphocytes and a high CD8+/regulatory T cell ratio are associated with favorable prognosis in ovarian cancer. Proc Natl Acad Sci USA.

[69] Galon J, Costes A, Sanchez-Cabo F, Kirilovsky A, Mlecnik B, Lagorce-Pagès C, Tosolini M, Camus M, Berger A, Wind P, Zinzindohoué F, Bruneval P, Cugnenc PH (2006). Type, density, and location of immune cells within human colorectal tumors predict clinical outcome. Science.

[70] Kryczek I, Banerjee M, Cheng P, Vatan L, Szeliga W, Wei S, Huang E, Finlayson E, Simeone D, Welling TH, Chang A, Coukos G, Liu R, Zou W (2009). Phenotype, distribution, generation, and functional and clinical relevance of Th17 cells in the human tumor environments. Blood.

[71] Zhao E, Maj T, Kryczek I, Li W, Wu K, Zhao L, Wei S, Crespo J, Wan S, Vatan L, Szeliga W, Shao I, Wang Y (2016). Cancer mediates effector T cell dysfunction by targeting microRNAs and EZH2 via glycolysis restriction. Nat Immunol.

[72] Nagarsheth N, Wicha MS, Zou W (2017). Chemokines in the cancer microenvironment and their relevance in cancer immunotherapy. Nat Rev Immunol.

[73] McMellen ME, Wakeman D, Erwin CR, Guo J, Warner BW (2010). Epidermal growth factor receptor signaling modulates chemokine (CXC) ligand 5 expression and is associated with villus angiogenesis after small bowel resection. Surgery.

[74] Ovrevik J, Refsnes M, Totlandsdal AI, Holme JA, Schwarze PE, Låg M (2011). TACE/TGF-α/EGFR regulates CXCL8 in bronchial epithelial cells exposed to particulate matter components. Eur Respir J.

[75] Øvrevik J, Holme JA, Låg M, Schwarze PE, Refsnes M (2013). Differential chemokine induction by 1-nitropyrene and 1-aminopyrene in bronchial epithelial cells: importance of the TACE/TGF-α/EGFR-pathway. Environ Toxicol Pharmacol.

[76] Fraser-Pitt DJ, Cameron P, McNeilly TN, Boyd A, Manson ED, Smith DG (2011). Phosphorylation of the epidermal growth factor receptor (EGFR) is essential for interleukin-8 release from intestinal epithelial cells in response to challenge with Escherichia coli O157 : H7 flagellin. Microbiology.

[77] Luppi F, Longo AM, de Boer WI, Rabe KF, Hiemstra PS (2007). Interleukin-8 stimulates cell proliferation in non-small cell lung cancer through epidermal growth factor receptor transactivation. Lung Cancer.

[78] Bolitho C, Hahn MA, Baxter RC, Marsh DJ (2010). The chemokine CXCL1 induces proliferation in epithelial ovarian cancer cells by transactivation of the epidermal growth factor receptor. Endocr Relat Cancer.

[79] Salcedo R, Martins-Green M, Gertz B, Oppenheim JJ, Murphy WJ (2002). Combined administration of antibodies to human interleukin 8 and epidermal growth factor receptor results in increased antimetastatic effects on human breast carcinoma xenografts. Clin Cancer Res.

[80] Son DS, Kabir SM, Dong YL, Lee E, Adunyah SE (2012). Inhibitory effect of tumor suppressor p53 on proinflammatory chemokine expression in ovarian cancer cells by reducing proteasomal degradation of IκB. PLoS One.

[81] Karki P, Hong P, Johnson J, Pajarillo E, Son DS, Aschner M, Lee EY (2017). Arundic Acid Increases Expression and Function of Astrocytic Glutamate Transporter EAAT1 Via the ERK, Akt, and NF-κB Pathways. Mol Neurobiol.

[82] Perez-Llamas C, Lopez-Bigas N (2011). Gitools: analysis and visualisation of genomic data using interactive heat-maps. PLoS One.

