# Supplementary material for: The TGFα-EGFR-Akt signaling axis plays a role in enhancing proinflammatory chemokines in triple-negative breast cancer cells
Source: Oncotarget. 2018 Jun 29;9(50):29286–303. doi: 10.18632/oncotarget.25389 (PMC6047672; doi:10.18632/oncotarget.25389)
Supplement: Supplementary file 1 [file oncotarget-09-29286-s001.pdf]

The TGFα-EGFR-Akt signaling axis plays a role in enhancing proinflammatory chemokines in triple-negative breast cancer cells

SUPPLEMENTARY MATERIALS

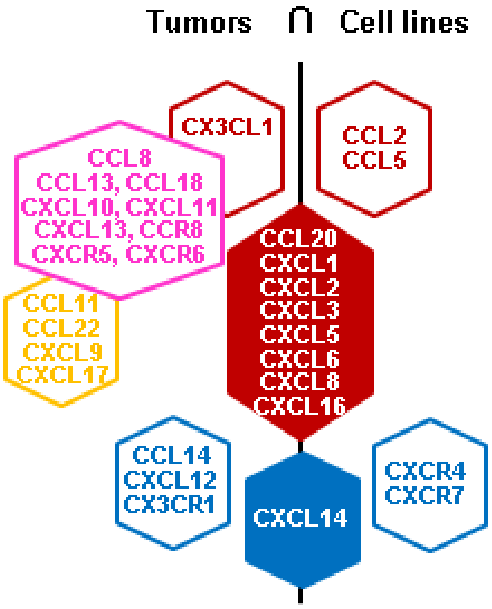

Supplementary Figure 1: Intersection between chemokine signatures in human BC cell lines and tissues. The intersection between the chemokine signatures in human BC cell lines and tissues (Figures 1 and 3) revealed that both TNBC cell lines and tumors expressed high levels of proinflammatory chemokines. Left and right panels list dominant chemokines in human BC tissues and cell lines, respectively. Red, yellow, blue colors represent TNBC, HER2 and LA subtypes, respectively. Pink color represents both BL and HER2 subtypes.

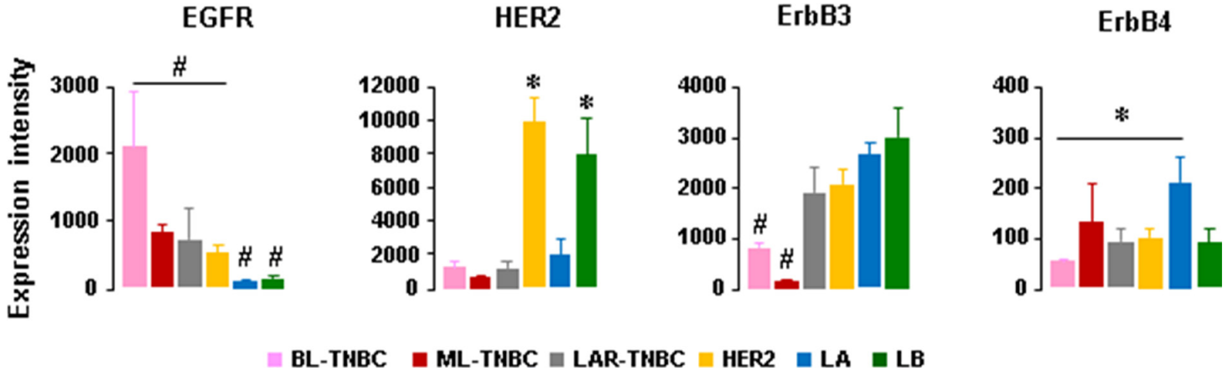

Supplementary Figure 2: Statistical analysis for the expression levels of EGFR family members in human BC cell lines. Pink, red, gray, yellow, blue and green bars indicate BL-TNBC, ML-TNBC, LAR-TNBC, HER2, LA and LB subtypes, respectively. The asterisk (*) and hash (#) indicate a statistically significant increase and decrease ($p \leq 0.05$) as determined by the ANOVA and Tukey's pairwise comparison, respectively. The Student's *t*-test were also performed for values marked with a horizontal line.

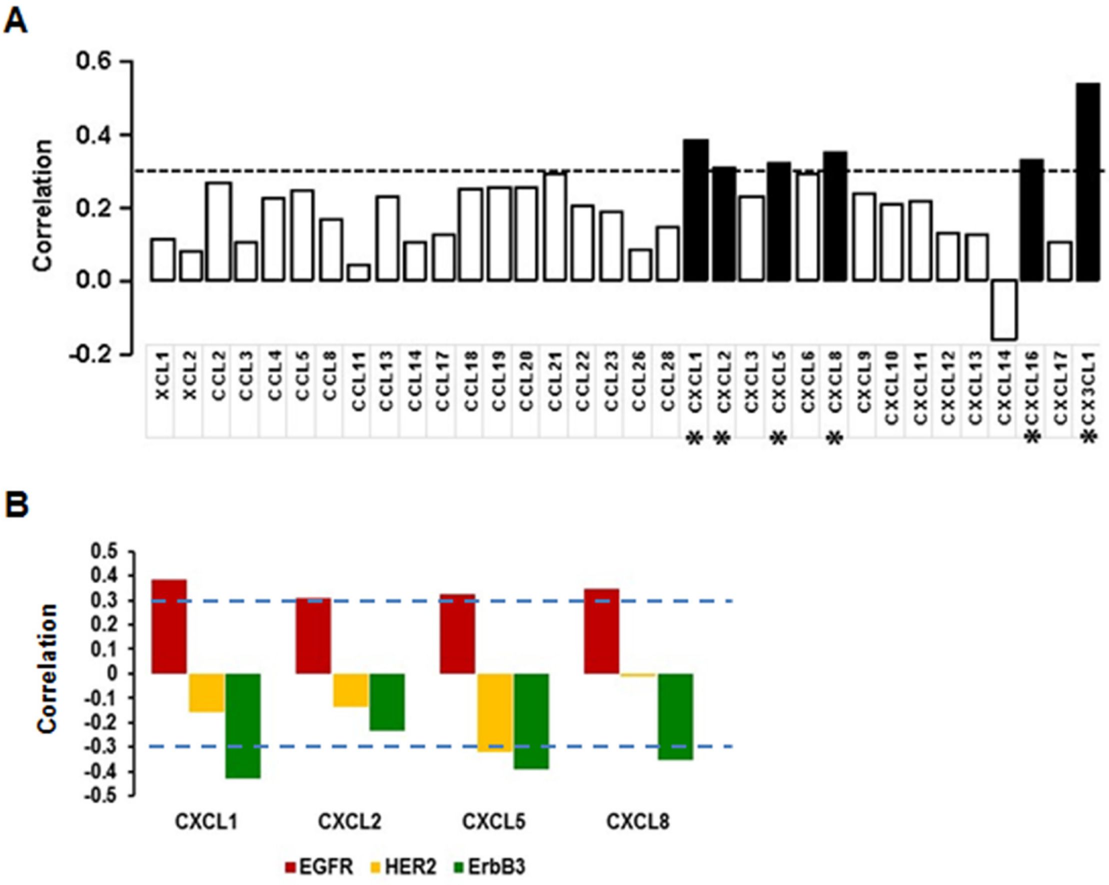

Supplementary Figure 3: Statistical analysis on the correlation between EGFR and chemokines in human BC tissues. (**A**) Correlation between EGFR and chemokine signature in human BC tissues from TCGA-based dataset. (**B**) Correlation between EGFR family and CXCL1, 2, 5 and 8 chemokines in human BC tissues from TCGA-based dataset. Red, yellow, and green bars indicate EGFR, HER-2 and ErbB3, respectively. The asterisk (*) indicates above 0.3 correlation (dot lines).

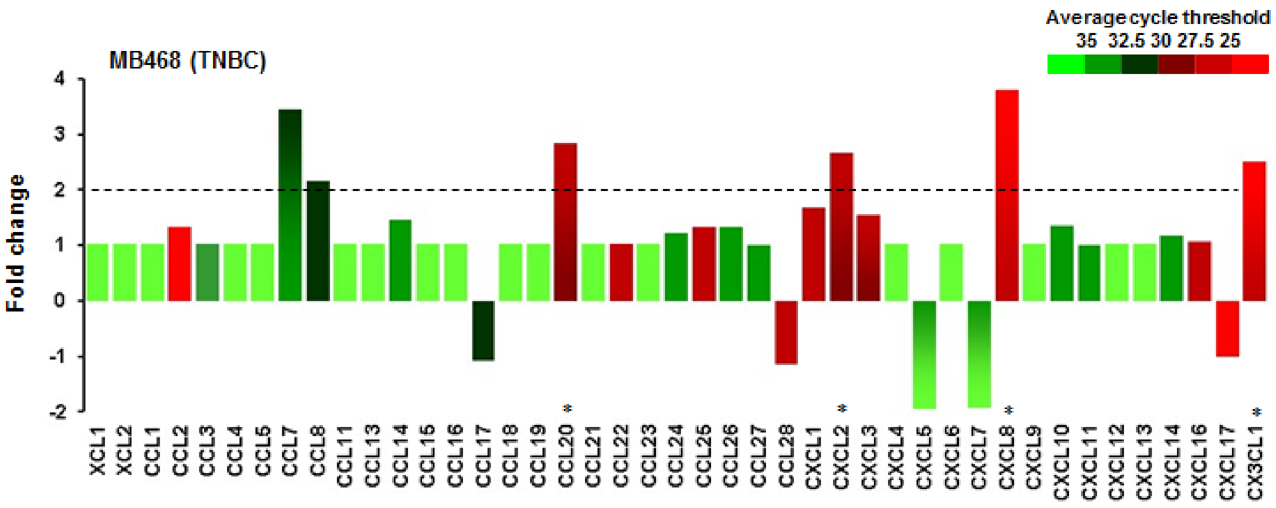

Supplementary Figure 4: EGF-induced chemokines in MB468 TNBC cells. Chemokine signatures in MB468 TNBC cells after a 1-h stimulation with recombinant human EGF (10 ng/ml) by a human chemokine PCR array. Chemokines with duplicate average cycle threshold of <30 are considered dominant. The asterisk (*) indicates increase that are larger than two-fold.

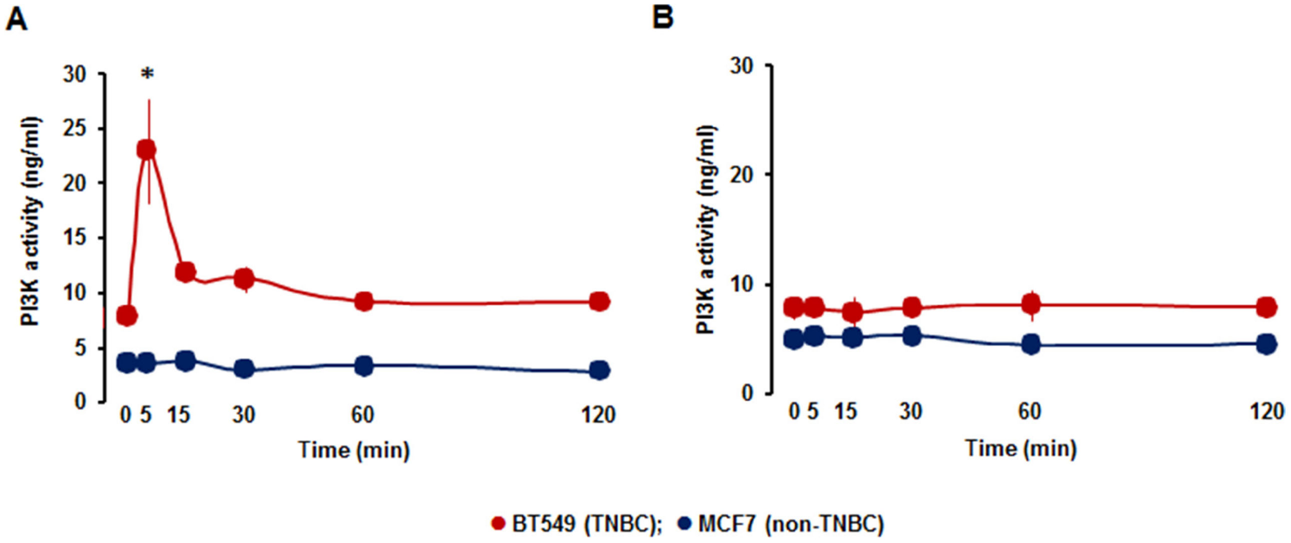

Supplementary Figure 5: EGF-induced PIK3CA activity in BT549 cells. (**A**) Effect of EGF on PIK3CA activity in non-TNBC (MCF7) and TNBC (BT549) cells after a time-dependent stimulation with recombinant human EGF (10 ng/ml) as revealed by a human PIK3CA ELISA. (**B**) Effect of TNF on PIK3CA activity in non-TNBC (MCF7) and TNBC (BT549) cells after a time-dependent stimulation with recombinant human TNF (10 ng/ml) as revealed by a human PIK3CA ELISA. The asterisk (8) indicates a statistically significant increase ($p \leq 0.05$) as determined by the ANOVA and Tukey's pairwise comparison, respectively.

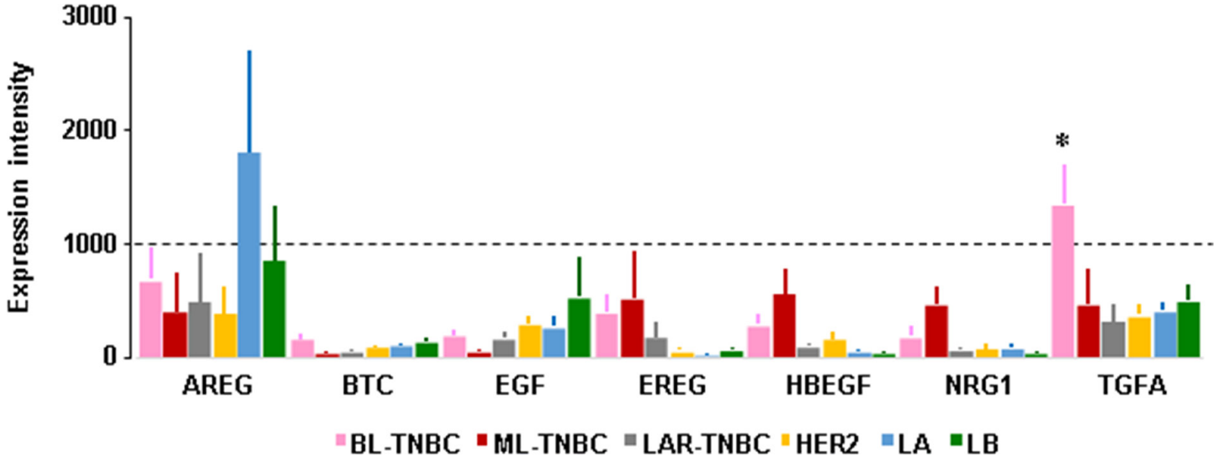

Supplementary Figure 6: Statistical analysis for EGFR-related ligands in human BC cell lines. Pink, red, gray, yellow, blue, and green bars indicate BL-TNBC, ML-TNBC, LAR-TNBC, HER2, LA and LB subtypes, respectively. The asterisk (*) indicates a statistically significant increase ($p \leq 0.05$) as determined by ANOVA and Tukey's pairwise comparison.
